# Tm4sf19 inhibition ameliorates inflammation and bone destruction in collagen-induced arthritis by suppressing TLR4-mediated inflammatory signaling and abnormal osteoclast activation

**DOI:** 10.1038/s41413-025-00419-y

**Published:** 2025-03-24

**Authors:** Sujin Park, Kwiyeom Yoon, Eunji Hong, Min Woo Kim, Min Gi Kang, Seiya Mizuno, Hye Jin Kim, Min-Jung Lee, Hee Jae Choi, Jin Sun Heo, Jin Beom Bae, Haein An, Naim Park, Hyeyeon Park, Pyunggang Kim, Minjung Son, Kyoungwha Pang, Je Yeun Park, Satoru Takahashi, Yong Jung Kwon, Dong-Woo Kang, Seong-Jin Kim

**Affiliations:** 1 GILO Institute, GILO Foundation, Seoul, Republic of Korea; 2 Medpacto Inc., Seoul, Republic of Korea; 3 Laboratory Animal Resource Center in Transborder Medical Research Center, Institute of Medicine, University of Tsukuba, Tsukuba, Japan; 4 Department of Biological Sciences, Sungkyunkwan University, Suwon, Republic of Korea; 5 Department of Anatomy and Embryology, Faculty of Medicine, University of Tsukuba, Tsukuba, Japan

**Keywords:** Bone, Diseases

## Abstract

Rheumatoid arthritis (RA) is an autoimmune disease characterized by inflammation and abnormal osteoclast activation, leading to bone destruction. We previously demonstrated that the large extracellular loop (LEL) of Tm4sf19 is important for its function in osteoclast differentiation, and LEL-Fc, a competitive inhibitor of Tm4sf19, effectively suppresses osteoclast multinucleation and prevent bone loss associated with osteoporosis. This study aimed to investigate the role of Tm4sf19 in RA, an inflammatory and abnormal osteoclast disease, using a mouse model of collagen-induced arthritis (CIA). Tm4sf19 expression was observed in macrophages and osteoclasts within the inflamed synovium, and Tm4sf19 expression was increased together with inflammatory genes in the joint bones of CIA-induced mice compared with the sham control group. Inhibition of Tm4sf19 by LEL-Fc demonstrated both preventive and therapeutic effects in a CIA mouse model, reducing the CIA score, swelling, inflammation, cartilage damage, and bone damage. Knockout of Tm4sf19 gene or inhibition of Tm4sf19 activity by LEL-Fc suppressed LPS/IFN-γ-induced TLR4-mediated inflammatory signaling in macrophages. LEL-Fc disrupted not only the interaction between Tm4sf19 and TLR4/MD2, but also the interaction between TLR4 and MD2. μCT analysis showed that LEL-Fc treatment significantly reduced joint bone destruction and bone loss caused by hyperactivated osteoclasts in CIA mice. Taken together, these findings suggest that LEL-Fc may be a potential treatment for RA and RA-induced osteoporosis by simultaneously targeting joint inflammation and bone destruction caused by abnormal osteoclast activation.

## Introduction

Rheumatoid arthritis (RA) is a chronic autoimmune disease that affects multiple joints, leading to irreversible joint damage, disability, and systemic bone loss.^1–3^ RA patients have a higher risk of developing osteoporosis than healthy people. Inflammatory cytokines such as TNF-α, IL-6 and IL-1, contribute to accelerated bone destruction.^1,4–6^ Under inflammatory conditions, proinflammatory cytokines released by macrophages induce RANKL expression on synovial fibroblasts.^7^ RANKL then binds to RANK on osteoclast precursor cells and promotes osteoclast maturation.^8,9^ TNF-α and IL-6 further contribute to osteoclast differentiation by directly targeting macrophages with permissive levels of RANKL and are also directly involved in bone remodeling in RA.^1^ Abnormal osteoclast activation in the synovium of RA patients leads to bone destruction, which is a major clinical problem in RA.^5^ Therefore, controlling both inflammation and bone destruction caused by abnormal osteoclast activation is important for effective RA treatment.^10^

Toll-like receptors (TLRs) are pattern recognition receptors that recognize pathogenic microorganisms and initiate the innate immune response.^11,12^ TLRs regulate inflammation and immune responses by activating the downstream signaling pathway NF-kB. TLRs are expressed in the inflamed joints of RA patients, where endogenous TLR ligands activate inflammatory signaling and joint bone destruction.^11,13^ Among TLRs, TLR4 expression is increased on peripheral blood monocytes and synovial fluid macrophages from RA patients and is also present in synovial tissues of RA patients.^11,14–17^ In an animal model of collagen-induced arthritis (CIA), TLR4 inhibition suppressed inflammatory cytokine induction and disease progression.^17^

Tetraspanins, also known as transmembrane 4 superfamily (TM4SF) proteins, contain multiple membrane-spanning domains with a short extracellular loop (SEL) domain and a large extracellular loop (LEL) domain and are involved in various cellular and physiological processes.^18^ The LEL domain of tetraspanins mediates interactions with tetraspanins, integrins, and other binding partners.^19^ Tm4sf19 (Transmembrane 4 L6 family member 19), a member of the TM4SF family, contributes to the progression of various diseases such as osteoporosis, arthrosclerosis, and obesity.^20–22^ However, the role of Tm4sf19 in the progression of rheumatoid arthritis-associated inflammation and bone destruction remains unclear.

In our previous study, we have demonstrated a critical role for Tm4sf19 in osteoclast differentiation. We have shown that deletion of the entire *tm4sf19* gene, deletion of only the genetic region encoding LEL in the *tm4sf19* gene, or treatment with a competitive inhibitor of Tm4sf19, LEL-Fc fusion (the large extracellular domain of Tm4sf19 fused to hIgG1), results in inhibition of osteoclast differentiation. In addition, we demonstrated that LEL-Fc administration suppressed osteoclast hyperactivation and thus bone loss in an ovariectomized mouse model.^22^

In this study, we showed that LEL-Fc treatment significantly reduced joint inflammation, cartilage destruction, and bone loss in mice with CIA. We also showed that LEL-Fc inhibited TLR4-mediated inflammatory signaling pathways and inflammatory M1 macrophage polarization. Collectively, our findings demonstrate that Tm4sf19 plays an important role in inflammation and osteoclast multinucleation in a mouse model of CIA. We propose that LEL-Fc may be a promising therapeutic agent targeting bone destructive diseases such as RA and RA-induced secondary osteoporosis.

## Results

### Expression of Tm4sf19 is increased in rheumatoid arthritis and in synovial macrophages and arthritis-associated osteoclastic macrophages (AtoM) from mice with CIA

**Fig. 1:**
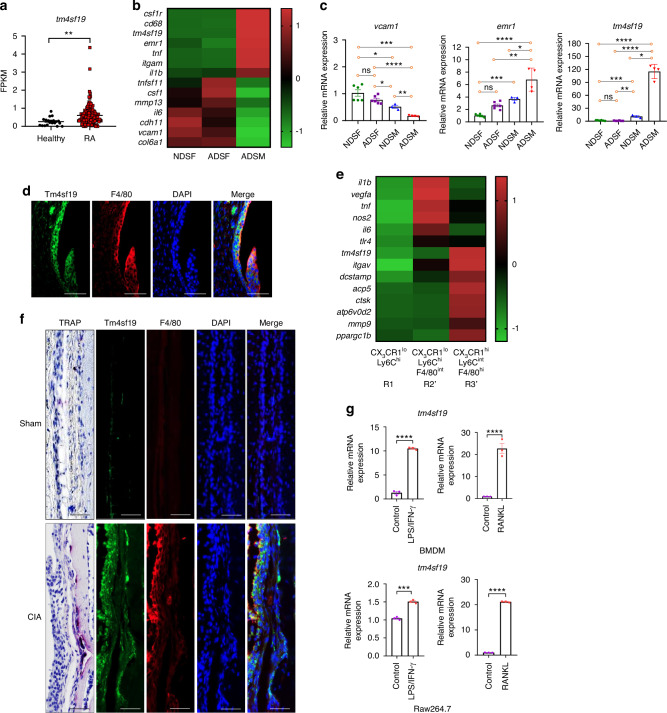
The expression of Tm4sf19 is increased in the whole blood of rheumatoid arthritis (RA) patients and the inflamed synovium of mice with collagen-induced arthritis. **a** Expression of *tm4sf19* in the whole blood of RA patients compared to healthy control was analyzed using a public data set (GSE120178). Relative expression of maker genes and *tm4sf19* in normal tissue-derived synovial fibroblasts (NDSF), arthritis tissue-derived synovial fibroblasts (ADSF), normal tissue-derived synovial macrophages (NDSM), and arthritis tissue-derived synovial macrophages (ADSM), was analyzed using a public data set (GSE142607) **b** and in primary culture **c**. **d** Representative images of immunofluorescence analysis of Tm4sf19 and F4/80. Bar indicates 50 μm. **e** Relative expression of marker genes and *tm4sf19* in CX_3_CR1^lo^Ly6C^hi^ (R1), CX_3_CR1^lo^Ly6C^hi^F4/80^int^ (R2’) and CX_3_CR1^hi^Ly6C^int^F4/80^hi^ (R3’, arthritis-associated osteoclastogenic macrophages, AtoM) cells, was analyzed using a public data set (GSE117149). **f** Representative images of co-immunostaining with Tm4sf19 and F4/80 in synovial macrophages and co-localization of Tm4sf19 and TRAP to the articular bones of sham and CIA mice. The bar indicates 50μm. **g** Relative mRNA expression of *tm4sf19* in inflammation condition and during osteoclast differentiation in macrophages. All the quantitative data were presented as mean ± SD, and the significance was calculated by student *t*-test or one-way ANOVA; *P < 0.05, **P < 0.01, ***P < 0.001, ****P < 0.000 1, ns=no significance

*tm4sf19* gene expression was increased in whole blood from patients with rheumatoid arthritis compared to healthy individuals (GSE120178) (Fig. 1a).^23^ Synovial fibroblasts and synovial macrophages are important therapeutic targets for RA treatment due to their role in inducing articular cartilage damage and articular bone destruction. Increased synovial macrophage infiltration often leads to articular bone destruction.^24^ From RNA-sequencing analysis dataset GSE142607, we found that the *tm4sf19* gene predominantly expresses in arthritis tissue-derived synovial macrophages (ADSM) (Fig. 1b).^25^ We confirmed that the expression of *vcam1*, was higher in fibroblast cells than macrophages and *emr1*, pan-macrophage marker, and *tm4sf19* was higher in macrophages than fibroblast cells in both normal and CIA. *tm4sf19* mRNA in ADSM was higher than in normal tissue-derived synovial macrophages (NDSM), in arthritis tissue-derived synovial fibroblasts (ADSF) and in normal tissue-derived synovial fibroblasts (NDSF) (Fig. 1c). Additionally, we observed that Tm4sf19 was predominantly expressed in macrophages in the sub-lining region of the synovium and colocalized with F4/80 in the inflamed synovium of mice with CIA compared to sham, healthy controls (Fig. 1d and Fig. S1). In inflamed joints of arthritis, macrophages have the potential to differentiate into osteoclast. Hasegawa et al. described the differentiation trajectory of osteoclasts in the inflamed synovium of arthritis and classified these cells into CX_3_CR1^lo^Ly6C^hi^ (R1), CX_3_CR1^lo^Ly6C^hi^F4/80^int^ (R2’) and CX_3_CR1^hi^Ly6C^int^F4/80^hi^ (R3’, arthritis-associated osteoclastogenic macrophages, AtoM) cells^26^. Their global transcriptome analysis revealed that R2’ cells express highly proinflammatory cytokines, whereas R3’ cells primarily express osteoclast differentiation markers. We confirmed that the expression of *tm4sf19* gene is increased in R2’ cells and further elevated in R3’ cells (Fig. 1e). Previously, we reported that Tm4sf19 plays an important role in osteoclast differentiation by interacting with integrin αv.^22^ In this study, we confirmed that the expression of *tm4sf19* was significantly increased in hyperactivated osteoclasts as evidenced by TRAP staining and in macrophages in synovium of CIA compared to sham, normal joints (Fig. 1f). In addition, immunofluorescence analysis of inflamed joints demonstrated that Tm4sf19 was colocalized with F4/80, a marker of macrophages (Fig. 1f). Additionally, we found that Tm4sf19 expression in bone marrow-derived macrophages and Raw264.7 macrophages was upregulated in response to LPS/IFN-γ-induced inflammation and M-CSF/RANKL-induced osteoclast differentiation (Fig. 1g). These data indicate that Tm4sf19 is involved in both inflammation and osteoclast differentiation in inflamed synovium.

### mLEL-Fc exhibits preventive therapeutic effects in a mouse model of collagen-induced arthritis

**Fig. 2:**
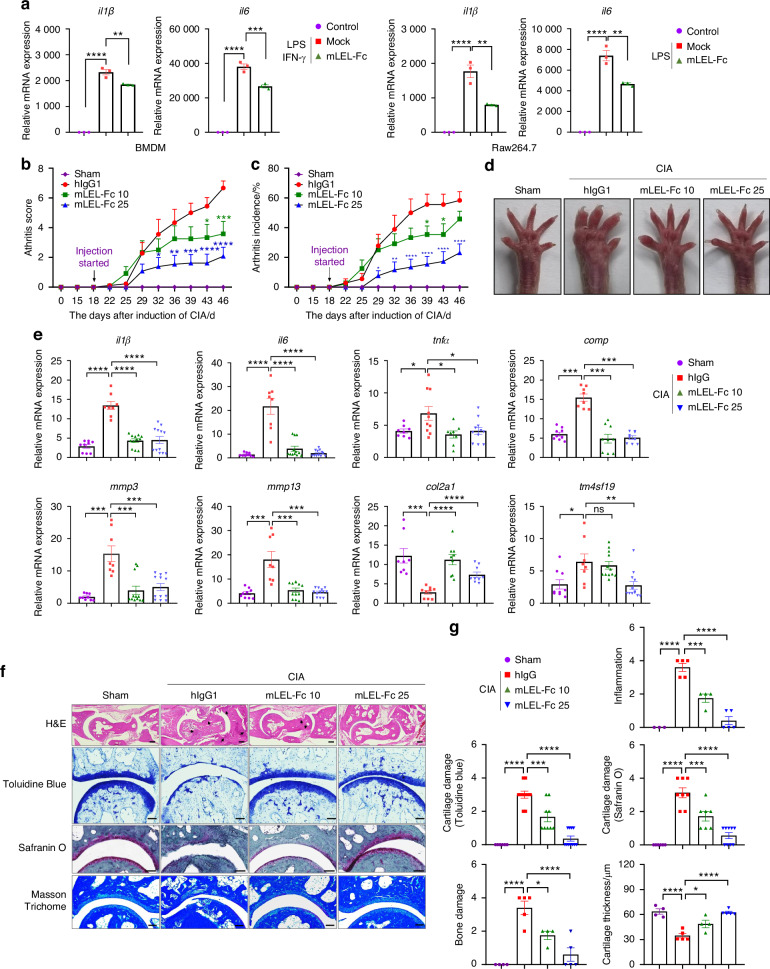
mLEL-Fc suppresses inflammation in mice with CIA. **a** The expression of inflammation markers was analyzed in macrophages in response to LPS/IFN-γ or LPS in BMDM and Raw264.7 cells. The effect of mLEL-Fc on the development of collagen-induced arthritis (CIA) was assessed by arthritis score (**b**), arthritis incidence (**c**). **d** Representative photographs of hind limbs 42 days after CIA-induction. Statistical analysis was performed with data from hIgG1 (*n* = 12), mLEL-Fc 10 mg/kg (*n* = 15) and mLEL-Fc 25 mg/kg (*n* = 15). **e** The expression of inflammation markers and genes involved in cartilage destruction of joint bones was analyzed by qRT-PCR. **f** Representative images of H&E, Toluidine Blue, Safranin O and Masson Trichome staining. Black arrows indicate bone damage in H&E. Articular cartilage thickness was measured using Masson Trichome staining. Scale bars represent 200 μm for H&E, 50 μm for Toluidine blue, and 100 μm for Safranin O staining and Masson Trichome staining. **g** Inflammation, bone damage, cartilage damages and cartilage thickness were analyzed. The significance was calculated by one-way ANOVA; *P < 0.05, **P < 0.01, ***P < 0.001, ****P < 0.000 1, ns=no significance

In our previous paper, we showed that treatment with mLEL-Fc fusion (the large extracellular domain of mouse Tm4sf19 fused to hIgG1), a competitive inhibitor of Tm4sf19, inhibited osteoclast differentiation.^22^ In this study, we investigated whether mLEL-Fc suppresses inflammation in macrophages and prevents cartilage damage in a mouse model of CIA. As shown in Fig. 1g, the expression of *tm4sf19* gene was induced by LPS/IFN-γ in macrophages. Next, we investigated whether mLEL-Fc treatment could suppress LPS/IFN-γ-induced inflammation in macrophages. Our results showed that mLEL-Fc effectively suppressed the expression of LPS/IFN-γ-induced inflammatory genes *il1β* and *il6* in BMDM, Raw264.7 and J774.A1 macrophages (Fig. 2a and Fig. S2). The fact that mLEL-Fc inhibits inflammation and osteoclast differentiation suggests that mLEL-Fc may have a potential to treat osteoclast-related bone destructive diseases such as RA. Therefore, we first investigated the therapeutic effect of mLEL-Fc in a mouse model of CIA, which mimics human RA. mLEL-Fc administration significantly reduced CIA scores and incidence in a dose-dependent manner compared with the hIgG1 control group (Fig. 2b, c). Treatment with mLEL-Fc alleviated erythema and swelling in the feet and ankles of CIA mice (Fig. 2d). In the CIA mouse model, the production of inflammatory cytokines and autoantibodies induces hyperdifferentiated osteoclasts, which induces bone destruction and inflammation. Therefore, we evaluated the expression of inflammation-related genes in the inflamed joints. The expression of *il1β*, *il6, tnfα*, and *comp* was elevated in the IgG treated group compared to sham group, but when 10 and 25 mg/kg mLEL-Fc were administered, the expression of these genes in the inflamed joints was significantly suppressed (Fig. 2e). mLEL-Fc treatment effectively suppressed the increased expression of *mmp3* and *mmp13*, which contributes to joint damage in CIA mice (Fig. 2e).^27^ In addition, mLEL-Fc treatment increased the expression of *col2a1* in the inflamed joints (Fig. 2e). Histological analysis using H&E staining confirmed that mLEL-Fc treatment suppressed inflammation and bone damage in mice with CIA. In addition, toluidine blue and safranin O staining showed that mLEL-Fc treatment effectively reduced cartilage destruction in a dose-dependent manner, highlighting a protective effect (Fig. 2f, g). In the hIgG1-treated group, articular cartilage thickness was reduced by 55.7% compared to the placebo group, but was recovered by 79.6% and 100% in the 10 mg/kg and 25 mg/kg mLEL-Fc-treated groups, respectively (Fig. 2f, g).

### hLEL-Fc showed therapeutic effects in mice with CIA

**Fig. 3:**
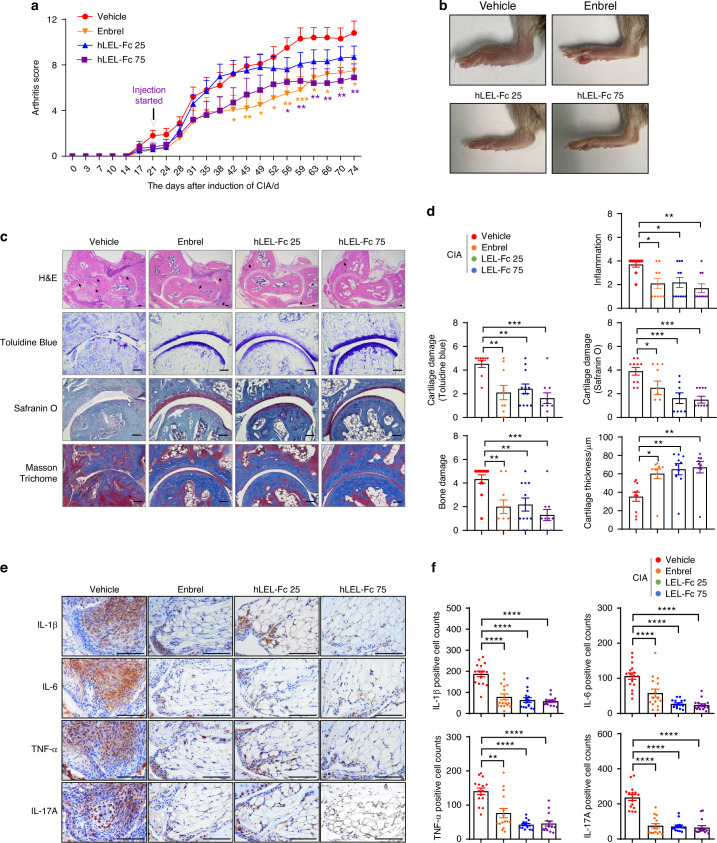
hLEL-Fc showed a therapeutic effect on CIA mouse model. **a** The effect of hLEL-Fc on CIA was assessed by arthritis score. **b** Representative images of hind limbs of CIA mice. **c** Representative photographs of H&E, Toluidine Blue, Safranin O and Masson Trichome staining. Scale bar indicates 100 μm for H&E and Masson Trichome staining, 50 μm for Toluidine blue, and Safranin O staining. Black arrows indicate bone damage in H&E. **d** Inflammation, bone damage, cartilage damage and cartilage thickness were analyzed from **c.** **e** Representative images of immunohistochemistry, examining inflammation makers. Scale bar indicates 100 μm. **f** Positive staining cells from the immunohistochemistry data **e** were analyzed. The significance was calculated by one-way ANOVA; *P < 0.05, **P < 0.01, ***P < 0.001, ****P < 0.000 1

After confirming the preventive effect of mLEL-Fc in mice with CIA, we investigated whether LEL-Fc also had therapeutic benefit. Treatment was initiated at disease onset with hLEL-Fc (human LEL region of Tm4sf19 fused to hIgG1, Fig. S3) or Etanercept (Enbrel, a human TNF receptor-IgG1 Fc fusion), a positive control for CIA treatment.^23^ To evaluate the therapeutic effect of hLEL-Fc, we administered the same 25 mg/kg dose of human LEL-Fc (hLEL-Fc) that was prophylactic with mouse LEL-Fc (mLEL-Fc) and a higher dose of 75 mg/kg, and found that the higher dose was significantly more effective in suppressing arthritis scores (Fig. 3a). However, the incidence of CIA was similar between the Enbrel- and hLEL-Fc-treated groups (Fig. S4). Although administration of hLEL-Fc did not suppress arthritis scores at the low dose, paw swelling, ankle joint inflammation, and bone damage analyzed by H&E staining were comparable to those observed in the high dose of hLEL-Fc and Enbrel treated group (Fig. 3a–c). Cartilage damage identified by toluidine blue and safranin O staining was significantly inhibited by both doses of hLEL-Fc and Enbrel treatment (Fig. 3c, d). Cartilage thickness, as measured by Masson Trichome staining, was increased in Enbrel and hLEL-Fc treated groups compared to Vehicle treated group (Fig. 3c, d). Immunohistochemical (IHC) staining results showed that the expressions of inflammatory markers IL-1β, IL-6, TNF-α, and IL-17A were decreased by both doses of hLEL-Fc and Enbrel treatment (Fig. 3e, f). These data indicate that LEL-Fc has potential in the treatment of rheumatoid arthritis.

### LEL-Fc treatment inhibits LPS/IFNγ-induced inflammatory signaling pathways by interfering with the interaction of Tm4sf19 with TLR4/MD2

**Fig. 4:**
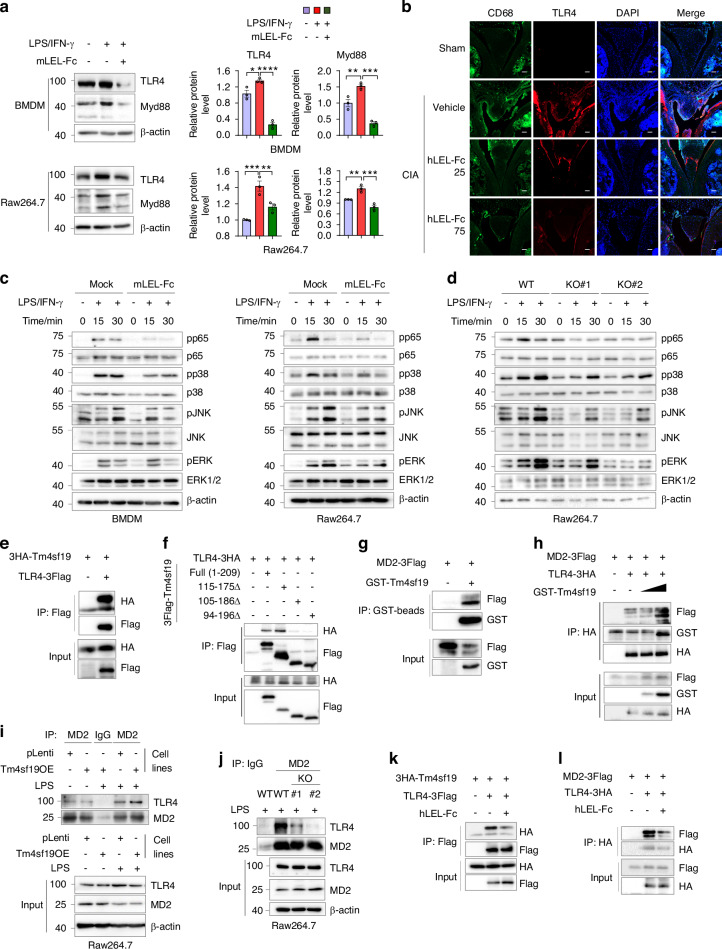
Tm4sf19 is involved in TLR4-related signaling in inflammation. **a** The effect of mLEL-Fc on LPS/IFN-γ-induced inflammatory TLR4 downstream signaling pathways was examined by western blotting with the indicated antibody. Relative protein expression was normalized by β-actin and quantified by ImageJ. **b** Representative immunofluorescence images of the synovium of CIA mice. Scale bar indicates 50 μm. Immunoblot analysis of NF-_K_B and MAPK signaling in respond to LPS/IFN-γ was performed in macrophages at the indicated time points, showing the effect of mLEL-Fc treatment (**c**) and deficiency of Tm4sf19 (**d**). **e** Immunoprecipitation results of Tm4sf19 and TLR4. **f** The interaction between TLR4 and WT and deletion mutants of Tm4sf19 was examined. **g** The interaction between Tm4sf19 and MD2 was confirmed by immunoprecipitation. **h** Immunoprecipitation results showed the effect of Tm4sf19 on TLR4/MD2 complex formation. The amount of GST-Tm4sf19 was gradually increased. **i** Immunoprecipitation results of TLR4/MD2 complex from pLenti (control) and Tm4sf19 expressing Raw264.7 cells with or without LPS treatment. **j** Endogenous interaction of MD2 and TLR4 in WT or Tm4sf19 knock out Raw 264.7 cell lines in the presence of LPS. The effect of hLEL-Fc treatment on the interaction between Tm4sf19 and TLR4 (**k**) or TLR4 and MD2 (**l**)

TLR4 signaling is a hallmark of inflammatory response.^15^ In this study, we demonstrated that LEL-Fc inhibited LPS/IFN-γ-induced inflammatory gene expression in BMDM and Raw264.7 macrophages (Fig. 2a). Furthermore, LEL-Fc inhibited LPS/IFN-γ-induced TLR4-related inflammatory protein expression in BMDM, Raw264.7, and J774.A1 macrophages (Fig. 4a and Fig. S5). Immunofluorescence analysis showed that hLEL-Fc treatment suppressed the expression of CD68, a macrophage marker, and TLR4 expression in the synovium of CIA mouse (Fig. 4b). In addition, LEL-Fc exhibited anti-inflammatory activity by inhibiting the phosphorylation of NF-kB and MAPK signaling pathways p65, p38, JNK, and pERK, which are downstream signaling pathways of TLR4 in BMDM and Raw264.7 macrophages (Fig. 4c and Fig. S6). To investigate the effect of Tm4sf19 deficiency on the inflammatory response, we generated Tm4sf19 knock-out Raw264.7 macrophages. In a previous study, we demonstrated that Tm4sf19 expression significantly increases during osteoclast differentiation.^22^ To demonstrate effective deletion of the Tm4sf19 protein, wild type and Tm4sf19 knockout clones of Raw264.7 cells were cultured with or without RANKL for osteoclast differentiation, and protein expression was confirmed by western blot. Expression of Nfatc1, a key regulator of osteoclast differentiation, was reduced in Tm4sf19 knockout clones in osteoclast differentiation (Fig. S7a). Next, we demonstrated that Tm4sf19 deficiency could suppress LPS/IFN-γ-induced inflammatory responses in Tm4sf19-deficient Raw264.7 macrophages and BMDMs derived from Tm4sf19KO mice (Fig. 4d, Fig. S7b and Fig. S8).

The fact that LEL-Fc suppresses LPS/IFN-γ-induced inflammation by downregulating TLR4 expression suggests that Tm4sf19 might bind to TLR4. Therefore, we investigated this possibility and confirmed that Tm4sf19 binds to TLR4 in transiently overexpressed cells (Fig. 4e). To determine the critical region of Tm4sf19 that interacts with TLR4, we utilized sequential deletion mutants of Tm4sf19.^22^ Interestingly, a mutant lacking the entire extracellular domain, amino acids 115–175, still interacted with TLR4, whereas a mutant lacking part of transmembrane 3, the extracellular domain, and part of transmembrane 4 (Tm4sf19_105-186Δ_) failed to interact (Fig. 4f). Moreover, a deletion mutant expressing only 120–169 amino acid of Tm4sf19 (Tm4sf19_120-169_) still interacted with TLR4, highlighting the importance of this region for the interaction (Fig. S9a). In addition, we found that Tm4sf19 interacted not only with TLR4 but also with MD2, a binding partner of TLR4 (Fig. 4g).^28^ We also confirmed the interaction between MD2 and Tm4sf19_120-169_ (Fig. S9b)_._ Next, we demonstrated that the endogenous interaction between Tm4sf19 and TLR4 in Raw264.7 cells stably expressing Tm4sf19 (Fig. S10a). Their endogenous interactions were increased by LPS treatment (Fig. S10b). Interestingly, we observed that the interaction between TLR4 and MD2 was enhanced by Tm4sf19 (Fig. 4h). In addition, we demonstrated that LPS treatment increased the interaction between TLR4 and MD2, and Tm4sf19 overexpression further enhanced their interaction (Fig. 4i). However, in the Tm4sf19 knockout cell line, the interaction between TLR4 and MD2 by LPS treatment was significantly inhibited (Fig. 4j). We also found that LEL-Fc inhibited not only the interaction between Tm4sf19 and TLR4, but also the interaction between TLR4 and MD2 (Fig. 4k, l). Interestingly, LPS treatment increased the interaction between TLR4 and MD2, but hLEL-Fc inhibited their interaction (Fig. S11). These findings suggest that Tm4sf19 contributes to the formation of the TLR4/MD2 complex, and LEL-Fc inhibits LPS/IFN-γ-induced inflammatory signals by inhibiting the formation of Tm4sf19/TLR4 or TLR4/MD2 complexes.

### LEL-Fc binds to M1-like macrophages and inhibits M1-mediated inflammatory signaling

It is known that synovial macrophages originate from circulating monocytes and resident macrophages in tissues. The balance between M1 macrophages, which produces pro-inflammatory signals, and M2 macrophages, which produces anti-inflammatory signals, is important for preventing and repairing tissue and bone destruction.^29^

**Fig. 5:**
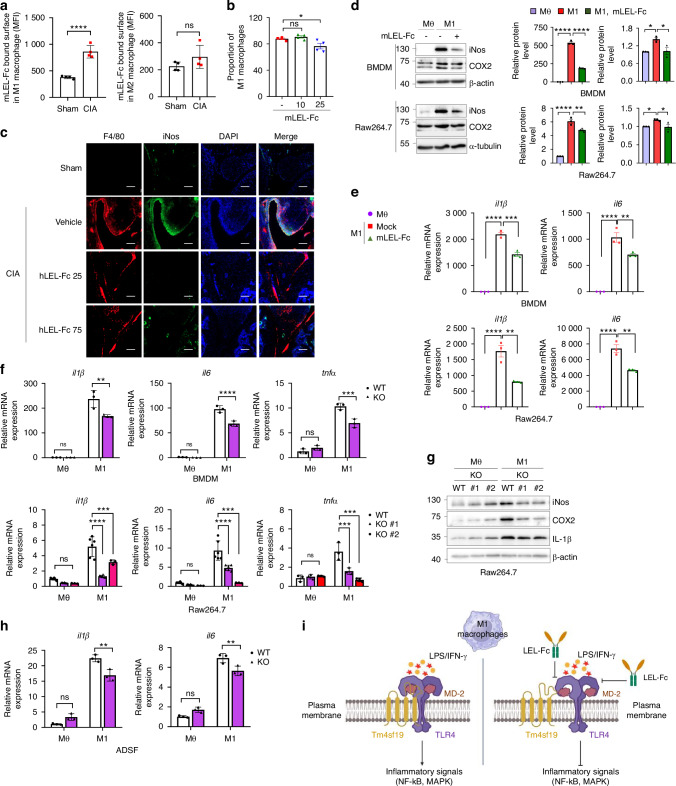
Tm4sf19 is involved in M1 macrophage polarization. FACS analysis of spleens from mice with CIA treated with hIgG1 or mLEL-Fc **a** Surface binding of LEL-Fc on M1 and M2 macrophages. **b** M1 macrophage polarization. **c** Representative immunofluorescence images of inflamed synovium in CIA mice with or without hLEL-Fc treatment. Scale bar indicates 50 μm. **d** Immunoblot analysis of M1 markers was performed after mLEL-Fc treatment. Relative protein expressions were calculated by normalization to β-actin protein expression. **e** The expression of M1 macrophage markers in response to mLEL-Fc was examined. **f** Relative mRNA expression of M1 marker genes in BMDM isolated from WT or Tm4sf19 KO mice or Tm4sf19 KO Raw264.7 cell lines. **g** Immunoblot analysis showed the effect of Tm4sf19 deficiency on M1 macrophage polarization. **h** qPCR analysis of inflammation marker genes in synovial fibroblast co-culture with M1 differentiated macrophages from WT or KO BMDM. **i** Proposed model for the inhibitory effect of LEL-Fc on Tm4sf19/TLR4 and TLR4/MD2 complex. Illustration was created in BioRender and modified. All the quantitative data were presented as mean ± SD and the significance was calculated by by student *t*-test, one-way ANOVA or two-way ANOVA; *P < 0.05, **P < 0.01, ***P < 0.001, ****P < 0.000 1, ns=no significance

To investigate the underlying mechanism by which LEL-Fc suppresses inflammation and bone destruction in mice with CIA, we examined the cell surface binding of LEL-Fc to various types of immune cells in the spleen. Interestingly, LEL-Fc binding to M1-like cell types (CD45^+^ CD11b^+^ F4/80^+^ MHC-II^+^ ) was increased in mice with CIA, but its binding to other immune cells, including M2-like cells, B cells and dendritic cells, were unchanged in arthritis induced mice (Fig. 5a and Fig. S12a). Then, we investigated the effect of LEL-Fc on the ratio of M1 and M2 macrophages, which are important for inflammatory signaling in arthritis. The proportion of M1-like macrophages (CD45^+^ CD11b^+^ F4/80^+^ MHC-II^+^ ) was decreased by 25 mg/kg mLEL-Fc administration in the spleen of mice with CIA (Fig. 5b and Fig. S12b). IL-17A released from T helper 17 cells plays an important role in autoimmune responses, increasing inflammation and damage in joint bones and promoting arthritis. Inhibiting IL-17A release by blocking Th17 activation can reduce arthritis. Therefore, we investigated whether LEL-Fc could reduce the proportion of CD45^+^ CD4^+^ IL-17A^+^ cells in the spleen of CIA mice. The proportion of CD45^+^ CD4^+^ IL-17A^+^ cells increased with collagen induction, but significantly decreased in the CIA group treated with LEL-Fc. However, there was no difference in the proportion of CD45^+^ CD4^+^ IFN-γ^+^ cells between the groups (Fig. S12c). In addition, LEL-Fc did not affect APC (DC1, DC2, and B) immune cells in the spleen of CIA mice (Fig. S12d). These data suggest that mLEL-Fc suppresses inflammatory responses by inhibiting the activation of Th17 cells through surface binding to M1-like macrophages. Immunofluorescence studies showed that the expression of macrophage markers F4/80 and iNOS was decreased in the inflamed synovium of CIA mice (Fig. 5c). We confirmed that LEL-Fc treatment inhibited the expression of iNOS and COX2, M1 macrophage markers, in BMDM and Raw264.7 macrophages (Fig. 5d). We also confirmed that LEL-Fc treatment suppressed the expression of M1 macrophage marker genes, including *il1β* and *il6* in BMDM and Raw 264.7 cells (Fig. 5e).

### Tm4sf19 deficiency inhibits the macrophage M1 polarization

To investigate whether Tm4sf19 is involved in macrophage polarization, we first examined the expression of marker genes *il1β*, *il6*, and *tnfα* induced by LPS/IFN-γ. The expression of M1 polarization marker genes was suppressed in BMDMs isolated from Tm4sf19 KO mice compared to wild-type (WT) BMDMs (Fig. 5f). In addition, the expression of these genes was significantly suppressed in Raw264.7 KO #1 and KO #2 cells lacking the Tm4sf19 gene compared to WT controls (Fig. 5f). We also demonstrated that the expression of M1 macrophage markers iNOS, COX2, and IL-1β is suppressed in KO M1 macrophages compared to WT M1 macrophages (Fig. 5g, Fig. S13, and Fig. S14a). However, the expression of the M2 macrophage marker Arg1 was similar between WT and KO macrophages (Fig. S14b).

In this study, we confirmed that Tm4sf19 is predominantly expressed in arthritis tissue-induced synovial macrophages (Fig. 1c). In synovial inflammatory conditions such as rheumatoid arthritis, activated synovial macrophages and synovial fibroblasts secrete proinflammatory cytokines such as IL-1, IL-6, and TNF-α. These cytokines induce osteoclastogenesis via RANKL secreted from synovial fibroblasts.^7^ Therefore, we next investigated the effects of macrophage M1 polarization on arthritis tissue-derived synovial fibroblasts in a co-culture system. We then examined the expression of inflammatory marker genes in arthritis tissue-derived synovial fibroblasts co-cultured with WT or KO M1 macrophages. Inflammatory markers in arthritis tissue-derived synovial fibroblasts co-cultured with KO M1 macrophages were suppressed compared to synovial fibroblasts co-cultured with WT M1 macrophages (Fig. 5h). These data indicated that inhibition of macrophage M1 polarization in KO macrophages suppresses inflammation in synovial fibroblasts and LEL-Fc suppresses proinflammatory cytokines by altering the ratio of M1/M2 macrophages and inhibiting the activation of M1 macrophages. Based on these results, we propose that inhibition of the interaction between Tm4sf19 and TLR4 and between TLR4 and MD2 by LEL-Fc results in the inhibition of LPS/IFN-γ-induced inflammatory signaling in M1 macrophages (Fig. 5i).

### LEL-Fc treatment suppressed bone damage in mice with CIA by blocking osteoclast hyperactivation

**Fig. 6:**
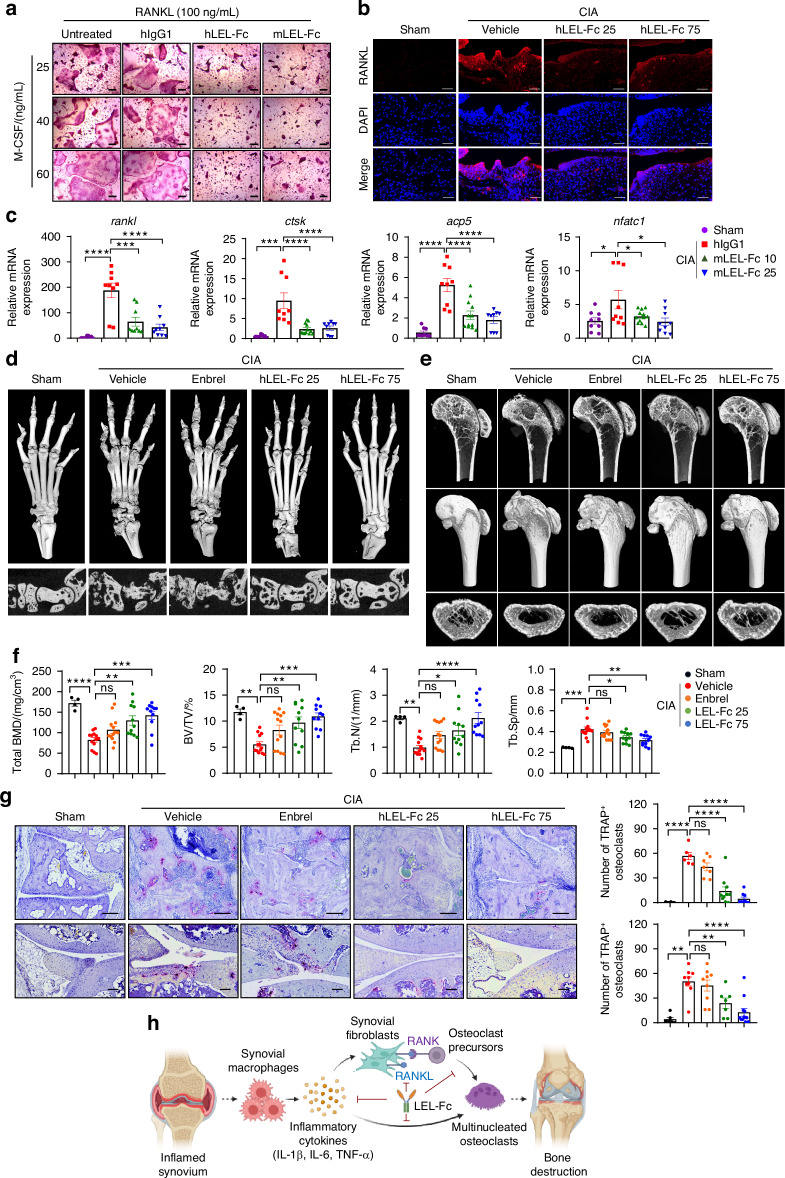
Treatment with LEL-Fc inhibits the activation of arthritis-associated osteoclastogenic macrophages in inflamed joints. **a** Representative image showing osteoclast multinucleation in untreated, hIgG1 treated, hLEL-Fc and mLEL-Fc treated osteoclasts in response to an increased amount of M-CSF confirmed by TRAP staining. Scale bar, 200 μm. **b** Representative images of immunofluorescence of the synovium of CIA mice treated with Vehicle or hLEL-Fc compared to the synovium of sham. Scale bar represents 50 μm. **c** Relative expression of *rankl* and osteoclast marker genes in the inflamed joints compared to sham using qRT-PCR. **d** Bone damages of mice CIA treated with Vehicle, Enbrel or hLEL-Fc was examined. μCT analysis of LEL-Fc treated bones of hind paw and ankle in CIA-induced mice. Representative μCT images (**e**) and trabecular bone analysis; BMD (bone mineral density), BV/TV (%) (bone volume/tissue volume), Tb.N (Trabucular number) and Tb.Sp (trabecular separation) (**f**). **g** TRAP staining analysis of ankle and inflamed synovium of CIA mice. Scale bar indicates 100 μm. **h** Schematic illustration of working model of the effect of LEL-Fc on arthritis. Illustration was created in BioRender and modified. All quantitative data are presented as mean ± SD, and significance was calculated using one-way ANOVA; *P < 0.05, **P < 0.01, ***P < 0.001, ****P < 0.000 1, ns=no significance

Our previous study revealed that LEL-Fc inhibits osteoclast differentiation in vitro and prevents bone destruction induced by hyperactivated osteoclasts in a mouse model of osteoporosis. We confirmed that both hLEL-Fc and mLEL-Fc effectively inhibits osteoclast differentiation even in the presence of high concentrations of M-CSF (Fig. 6a and Fig. S15). Danks et al. demonstrated that RANKL is predominantly expressed in synovial fibroblasts and plays a key role in osteoclast formation and bone erosion during arthritis-associated joint inflammation.^7^ Based on this, we investigated whether LEL-Fc inhibits RANKL expression in synovial fibroblast of CIA mice. Immunofluorescence analysis revealed that RANKL positive cells were present in the synovium of CIA mice, and treatment of LEL-Fc effectively suppressed RANKL expression (Fig. 6b). Furthermore, we confirmed that the elevated *rankl* expression in inflamed joints was significantly suppressed by LEL-Fc treatment (Fig. 6c). These findings suggest that LEL-Fc may inhibit CIA-induced RANKL production in the synovium. Serum RANKL levels were slightly increased in CIA mice treated with hIgG1 and decreased in CIA mice treated with LEL-Fc compared to the sham group. However, the difference in serum RANKL levels between the groups was not statistically significant (Fig. S16). It is known that the production of inflammatory cytokines and autoantibodies induces hyperdifferentiated osteoclasts that cause bone destruction and inflammation in the CIA model. Therefore, we investigated whether LEL-Fc could inhibit abnormal osteoclast differentiation activated by inflammatory cytokines in joints and long bones. First, we found that the expression of osteoclast differentiation marker genes *ctsk*, *acp5*, and *nfatc1* was increased in the inflamed joint of CIA mice due to abnormal osteoclast activation, but this was suppressed by administration of mLEL-Fc (Fig. 6c). μCT analysis showed that LEL-Fc treatment significantly inhibited the destruction of joint bones, including the hind paw and ankle compared with the vehicle control group (Fig. 6d and Fig. S17a).^30^ However, Enbrel failed to inhibit CIA-induced bone loss (Fig. 6d). Next, μCT analysis of the femur showed that the decreases in total bone mineral density (BMD), bone volume/tissue volume (BV/TV)/%, and trabecular numbers caused by CIA were restored by administrating 25 mg/kg LEL-Fc and further improved by 75 mg/kg LEL-Fc treatment (Fig. 6e, f and Fig. S17b and c). In addition, the hyperactivated osteoclasts, identified by TRAP staining, which led to bone destruction, were significantly suppressed by LEL-Fc in the ankle, synovium and femur of CIA mice (Fig. 6g and Fig. S18). These data indicated that LEL-Fc treatment effectively suppressed osteoclast-mediated pathologic bone erosion in inflamed joints in the CIA mouse model (Fig. 6, Fig. S17 and Fig. S18). These data suggest that LEL-Fc suppresses bone loss in inflammatory arthritis by blocking inflammation-induced osteoclast hyper-differentiation and resulting bone resorption by suppressing inflammation and RANKL production (Fig. 6h).

## Discussion

In inflammatory diseases such as osteoporosis and rheumatoid arthritis, bone metabolism is imbalanced due to the activation of pro-inflammatory cytokines such as IL-1β and TNF-α.^2,3,23^ These cytokines stimulate the induction of RANKL and ICAM-1 on synovial fibroblast, leading to osteoclast maturation.^7,30–33^ Immune stimuli from cytokines, lymphocytes, and macrophages are also involved in bone metabolism.^34^ Cross-communication between osteocytes and immune cells mediates bone remodeling processes in inflammatory bone diseases.^35^ The development of CIA involves an immune cell response that causes joint inflammation, swelling, and bone metabolic imbalance by macrophages and neutrophils infiltrating the synovium, resulting in bone destruction.^30,36^ In a previous study, we showed that LEL-Fc administration suppressed bone loss induced by estrogen deficiency.^22^ In this study, we showed that LEL-Fc effectively suppressed inflammatory immune responses and bone metabolism that favor bone resorption (Figs. 2, 3 and 6). These data suggest that Tm4sf19 may play an essential role in crosstalk between bone cells and immune cells, indicating that Tm4sf19 may be a novel therapeutic target for inflammatory bone diseases.

TLR4 plays a crucial role in the innate immune response by activating the inflammatory responses and inducing ECM degradation, which leads to tissue destruction and bone damage in the inflamed synovium of RA. TLR4 is mainly expressed on synovial macrophages, fibroblasts, endothelial cells, and dendritic cells, which produce inflammatory cytokines and chemokines in the inflamed joints of RA. TLR4 inhibitors suppressed joint inflammation in adjuvant-induced arthritis.^15,37^ Various approaches have been reported to inhibit the inflammatory signaling of TLR4, such as miRNA technology, neutralizing anti-TLR4 antibodies, small molecules, and MD2 inhibitors.^16^ However, since TLR4 plays a critical role in host defense by recognizing pathogen-associated molecular patterns (PAMPs) and damage-associated molecular patterns (DAMPs), TLR4 inhibition may increase susceptibility to infections.^16^ In this study, we found that both Tm4sf19 deficiency and inhibition of Tm4sf19 activity suppressed LPS/IFN-γ-induced expression of TLR4 and its downstream signaling pathways (Fig. 4a–d, Fig. S5–S8). Furthermore, we showed that the interaction between Tm4sf19 and TLR4 occurred through the LEL domain of Tm4sf19, and that this binding was inhibited by LEL-Fc treatment (Fig. 4k). These data suggest that LEL-Fc can suppress synovial inflammation in mice with CIA by blocking the TLR4-mediated signaling pathway through interfering with the interaction between Tm4sf19 and TLR4. Among the Toll-like receptor (TLR) family, TLR1-9 are expressed on osteoclast precursor cells.^12^ TLR4 is known to be involved in osteoclast formation, growth, differentiation, survival, and bone resorption.^38,39^ TLR4 deficiency suppresses bone loss by inhibiting RANKL expression.^38–40^ Blockade of the TLR-4/MyD88/NF-kB signaling pathway inhibited osteoclastogenesis.^41–44^ Therefore, our data suggest that increased expression of Tm4sf19 protein in synovial macrophages in rheumatoid arthritis amplifies TLR4-mediated inflammatory signals, thereby worsening rheumatoid arthritis.

In our previous study, we demonstrated that Tm4sf19 regulates osteoclast differentiation by interacting with integrin αv, and LEL-Fc suppressed the osteoclastogenesis by inhibiting their interaction.^22^ The expression of integrin αvβ3 was increased in RA synovium compared to healthy controls, and integrin αvβ3 was highly expressed in synovial fibroblasts involved in cell migration, adhesion, and invasion. It promotes synovial hyperplasia and pannus formation through inflammatory responses in RA synovium. Integrin αvβ3 was also expressed in the synovial lining, where it activated inflammation and tissue remodeling.^45^ It was mainly expressed in osteoclasts and endothelial cells in inflamed synovium. Targeting integrin αvβ3 with small molecules and antibodies reduced synovial inflammation and bone destruction, suggesting that integrin αvβ3 is a potential therapeutic target for RA.^45^ These data suggested that LEL-Fc may have synergistic therapeutic effects on RA by simultaneously targeting integrin αvβ3 and TLR4 signaling to suppress the production of inflammatory cytokines and chemokines, thereby alleviating inflammatory responses, severe tissue destruction, and bone damage.

In the synovium of RA patients, M1 macrophages activated by TLR and IFN signaling produce inflammatory cytokines that promote tissue and cartilage damage and osteoclast differentiation, resulting in bone erosion and joint destruction.^14^ Meanwhile, activated M2 macrophages produce anti-inflammatory cytokines that contribute to the remission in RA patients. The imbalance between M1 and M2 macrophages contributes to the inflammatory progression of RA.^29,46^ In this study, we showed that Tm4sf19 is mainly involved in M1 inflammatory signaling. This was confirmed by the results that LPS/IFN-γ-induced inflammation was suppressed by both LEL-Fc treatment and Tm4sf19 knockout (Fig. 5). MAPK signaling pathways, including phosphorylation of JNK, p38, and pERK1/2, are activated by M1 macrophage-induced inflammation and cytokines TNF-α, IL-1β, and IL-6 in the synovium of RA patients. In addition, these inflammatory cytokines in synovial macrophages activate inflammatory signaling through the TLR4-induced NF-κB signaling pathway. We demonstrated that LEL-Fc effectively inhibits LPS/IFN-γ-induced MAPK and NF-κB signaling in BMDM and Raw264.7 macrophages (Fig. 4c and d).

An ideal treatment strategy for rheumatoid arthritis would be to control inflammation and prevent bone destruction by inhibiting excessive osteoclast activation.^6,47^ Several studies discuss treating arthritis with drugs that have dual roles in regulating inflammation and osteoclast activity, either directly or indirectly.^48–51^ For example, TNF-α, JAK, or IL-6 inhibitors directly suppress inflammation but indirectly suppress osteoclast activation.^51^ Similarly, bisphosphonate and RANKL inhibitors directly block osteoclast activation but indirectly inhibit inflammation.^48,52^ Among the inflammatory cytokines in RA, TNF-α may contribute to osteoclast differentiation, as anti-TNF-α treatment reduces both inflammation and bone destruction in RA. TNF-α also targets macrophages together with RANKL to induce osteoclast differentiation. Despite significant advances in the treatment of RA with tumor necrosis factor (TNF) inhibitors and JAK kinase inhibitors (JKIs), only 20%–30% of patients experience remission.

Here, we demonstrate that LEL-Fc directly suppresses inflammation and osteoclast activation. LEL-Fc blocks arthritis progression and bone destruction primarily by inhibiting inflammatory macrophage polarization, and the inflammatory response and RANKL expression in synovial fibroblast in joint inflammation, and subsequently inhibiting osteoclast precursor differentiation and osteoclast maturation associated with arthritis. This activity is attributed to the unique function of Tm4sf19, which is expressed exclusively in activated macrophages and differentiated osteoclasts, and specifically influences macrophage-driven-inflammation and osteoclast multinucleation. These data suggest that Tm4sf19 is a potential therapeutic target for rheumatoid arthritis and RA-mediated secondary osteoporosis.

## Materials and methods

### Mice

All animal experiments were approved by the Institutional Animal Care and Use Committee (IACUC) of Medpacto (Approval No. 2021-0009) or Catholic University (Approval No. CUMC-2023-0067-01) and followed the ARRIVE 2.0 guidelines.

### Collagen-induced arthritis mouse model

8-week-old DBA/1 mice were used for CIA induced mouse model after 3 weeks of resting period. CIA was induced by immunization with type II collagen/CFA (complete Freund’s Adjuvant) emulsion (Hooke Lab, EK-0210 or Chondrex 200-11) at day 0 and boosted at day 18 or 21 with collagen/IFA (Incomplete Freund’s Adjuvant) emulsion (Hooke Lab, EK0211, Chondrex, 700-2) between the ventral and lateral vein of the tail. Started at day 15 (for prophylactic therapeutic effect) or day 24 (for treatment effect) or hIgG1-Fc or LEL-Fc was i.v. or s.c. administrated twice a week until the end of the experiment. The groups were divided after measuring the level of CII-specific IgG2a in serum. Enbrel (Etanercept, Pfizer, FY5734), a positive control, was administrated three times a week s.c. Mice were scored for clinical signs of arthritis followed the guideline of Hooke laboratory.

### Preparation of mouse bone marrow cells

Mouse bone marrow cells (BMs) were obtained from tibias and femurs of 6 to 10-week-old wild type or *tm4sf19*^−/−^ *knock-out*^22^ mice by flushed with DMEM (High glucose, WelGENE Inc., Daegu, Korea) culture media. Bone marrow derived macrophages (BMDMs) were obtained by incubating BMs with 25 ng/mL for 7 days and treated with LPS/IFN-γ or il4 for the further experiment. BMs were incubated with 5 ng/mL M-CSF in α-MEM overnight and non-adherent cells were incubated for 3 days in 30 ng/mL M-CSF to obtain bone marrow macrophages (BMMs). For in vitro osteoclastogeneisis, BMMs were incubated with M-CSF and RANKL until multinucleated osteoclast was shown.

### Isolation of mouse primary synovial cells

Mouse primary synovial fibroblasts (SFs) and macrophages (SMs)were isolated from synovitis tissues in a mouse with CIA as followed previous report.^25^ Synovitis tissues were digested with collagenase type IV (Sigma, USA) in DMEM with 10% fetal bovine serum (FBS) and 1% antibiotic-antimycotic solution. Filtered the cell suspension through a 40 μm cell strainer. To obtain SFs, cells were seeded on the collagen-coated dishes for 1 h and non-adherent cells were transferred to collagen-non-coated plated. Adherent cells were cultured in fresh medium and sub-cultured fewer than 5 passages to obtain purified fibroblast. Transferred non-adherent cells including SMs and SFs were incubated for 1 day and non-adherent cells were removed and washed 2 or 3 times. To obtain SMs, adherent cells were cultured for 1 ~ 2 weeks in culture medium and detached SFs by treated with 0.05% trypsin in HBSS. After removed detached cells, fresh medium was added to expand SMs.

### Micro-CT analysis

After fixed femurs with 10% neutral-buffered formalin, micro-computed tomographic analysis was performed with Skyscan 1171 (50 kV, 200 μA, 600 ms). After scan data was reconstructed by NRecon software, trabecular morphometry analysis was performed by CTAn software and visualized by CTvox software. For bone analysis, trabecular structures 2 mm away from the growth plate were selected.

### Histological analysis

Fixed hind limbs were decalcified and paraffin embedded. For evaluation inflammation, bone damage and cartilage damage, serial sections were stained with Hematoxylin and Eosin, Toluidine blue, Safranin O, Masson Trichome or TRAP (COSMO BIO, PMC-AK04-COS). For immunohistochemical staining, serial sections were incubated with indicated antibodies followed by incubated with HRP-secondary antibody. To access inflammation, four different fields were captured from the inflamed synovium and positive cells were counted using ImageJ software (Wayne Rasband, NIH, USA). For immunofluorescence staining, serial sections were incubated with indicated antibodies followed by Alexa Fluor 488 or 647 antibodies. Images were captured using confocal microscopy (Zeiss, LSM800).

### Tm4sf19 knock-out cell generation

Tm4sf19 knock-out in Raw264.7 cells were generated by CRISPR/CAS9 system. Synthesized the guide RNA by Precision gRNA Synthesis Kit (A29377, Invitrogen) and TrueCutTM Cas9 Protein V2 (A36496, Invitrogen) were electroporated into the cell. After single cell selection, the deletion was confirmed through PCR using genomic DNA.

### Immunoprecipitation and immunoblot analysis

Cells were lysed with RIPA (50 mmol/L Tris-HCL, pH 7.5/ 150 mmol/L NaCl, 1% NP-40/0.5% sodium deoxycholate/0.1% SDS) supplemented protease inhibitors (Roche, 11873580001) and phosphatase inhibitor (Roche, 04906831001). For immunoblot analysis, proteins from whole cell lysates were boiled with Laemini sample buffer, separated in SDS-PAGE gel, transferred to PVDF, incubated with indicated antibodies followed by HRP-conjugated secondary antibody and detected using chemiluminescent ECL solution (BIOMAX). For immunoprecipitation, whole cell lysates were incubated with the indicated antibody overnight followed by incubation of protein G dynabeads (Invitrogen, 10004D). Dynabeads were washed three times, boiled with Laemini sample buffer. Immunoprecipitated proteins were analyzed by western blot with indicated antibodies and detected. Relative protein expression was quantified by ImageJ.

### Real-time quantitative PCR (RT-PCR) analysis

Total RNA was extracted from joint bones, BMDMs, Raw264.7 cells, using Easyblue (Intron, 17061). cDNA was synthesized from 0.5~ 2 μg of total RNA and target gene expression was analyzed by Q-PCR using a QuantStudio (Applied Biosystems).

### ELISA assay

Serum RANKL concentration was determined by mouse RANKL ELISA kit (R&D Systems, MTR00) as followed manufacturer’s instruction.

### Statistical analysis

Statistical data analysis was performed by student *t*-test, one-way ANOVA or two-way ANOVA using GraphPad Prism 8 software. The significance was presented on the graphs as *P < 0.05, **P < 0.01, ***P < 0.001, ****P < 0.000 1, ns=no significance.

## Supplementary information

Supplementary Figures

Supplemental materials and methods

nr-reporting-summary
