# Supplementary material for: Tm4sf19 inhibition ameliorates inflammation and bone destruction in collagen-induced arthritis by suppressing TLR4-mediated inflammatory signaling and abnormal osteoclast activation
Source: Bone Res. 2025 Mar 24;13:40. doi: 10.1038/s41413-025-00419-y (PMC11933450; doi:10.1038/s41413-025-00419-y)
Supplement: Supplementary file 1 — Supplementary Figures [file 41413_2025_419_MOESM1_ESM.docx]

**Tm4sf19 inhibition ameliorates inflammation and bone destruction in collagen-induced arthritis by suppressing TLR4-mediated inflammatory signaling and abnormal osteoclast activation**

Sujin Park^1^, Kwiyeom Yoon^2^, Eunji Hong^1^, Min Woo Kim^2^, Min Gi Kang^1^, Seiya Mizuno^3^, Hye Jin Kim^2^, Min-Jung Lee^2^, Hee Jae Choi^2^, Jin Sun Heo^1^, Jin Beom Bae^2^, Haein An^1^, Naim Park^2^, Hyeyeon Park^1,4^, Pyunggang Kim^1^, Minjung Son^1,4^, Kyoungwha Pang^1^, Je Yeun Park^1^, Satoru Takahashi^5^, Yong Jung Kwon^1^, Dong-Woo Kang^2^, and Seong-Jin Kim^1,2*^

^1^GILO Institute, GILO Foundation, Seoul, Republic of Korea, ^2^Medpacto Inc., Seoul, Republic of Korea, ^3^Laboratory Animal Resource Center in Transborder Medical Research Center, Institute of Medicine, University of Tsukuba, Tsukuba, Japan, ^4^Department of Biological Sciences, Sungkyunkwan University, Suwon, Republic of Korea, and ^5^Department of Anatomy and Embryology, Faculty of Medicine, University of Tsukuba, Tsukuba, Japan.

**Supplementary Figures**

**
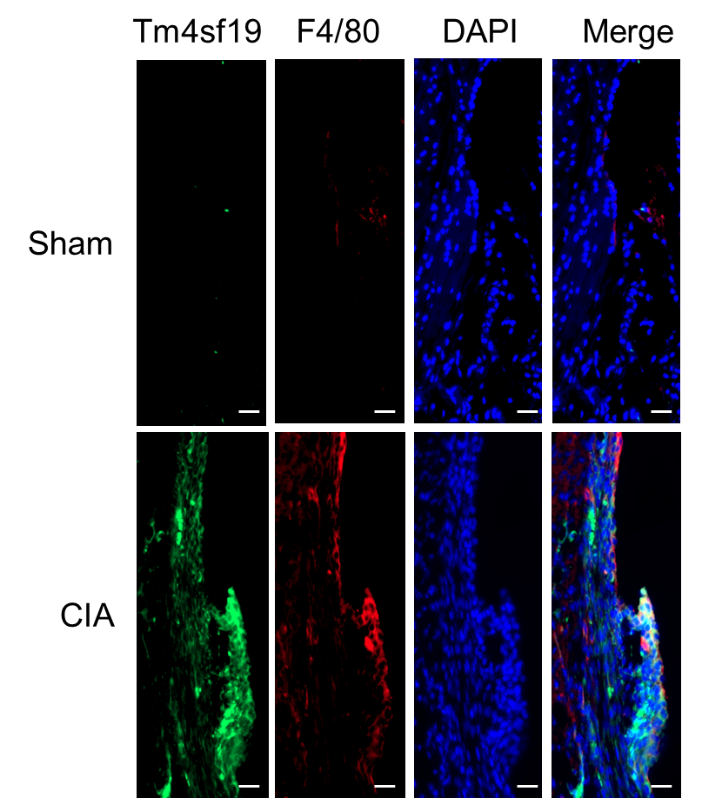
**

**Figure S1. Tm4sf19 expression in synovium of CIA.** Representative images of immunofluorescence analysis of Tm4sf19 and F4/80 in synovium of sham or CIA mice. The bar indicates 20μm.

**
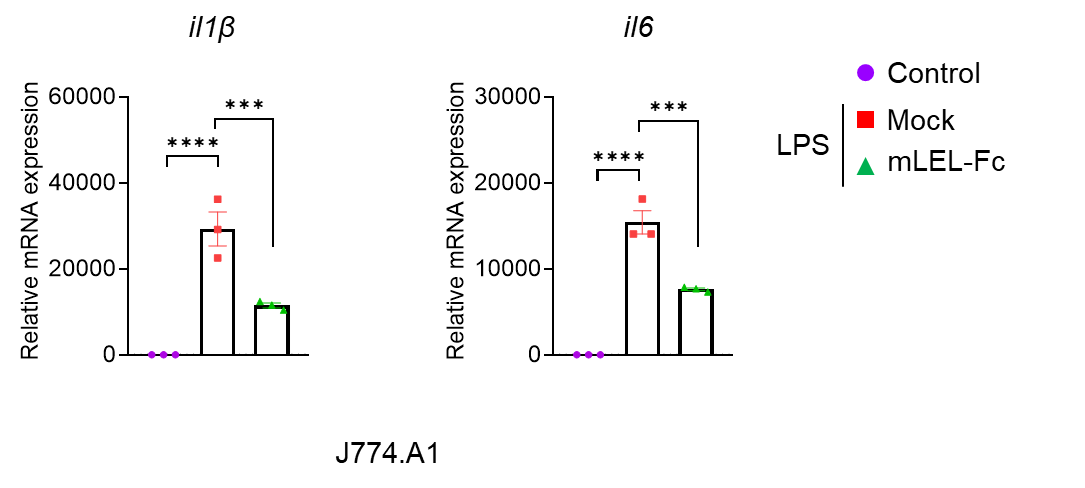
**

**Figure S2. mLEL-Fc suppresses LPS-induced inflammatory signaling.** Representative data show the inflammatory TLR4 downstream signaling pathways in J774 macrophage cells. All quantitative data are expressed as mean ± SD, and significance was calculated using one-way ANOVA; ***p <0.001, ****p <0.0001.

**
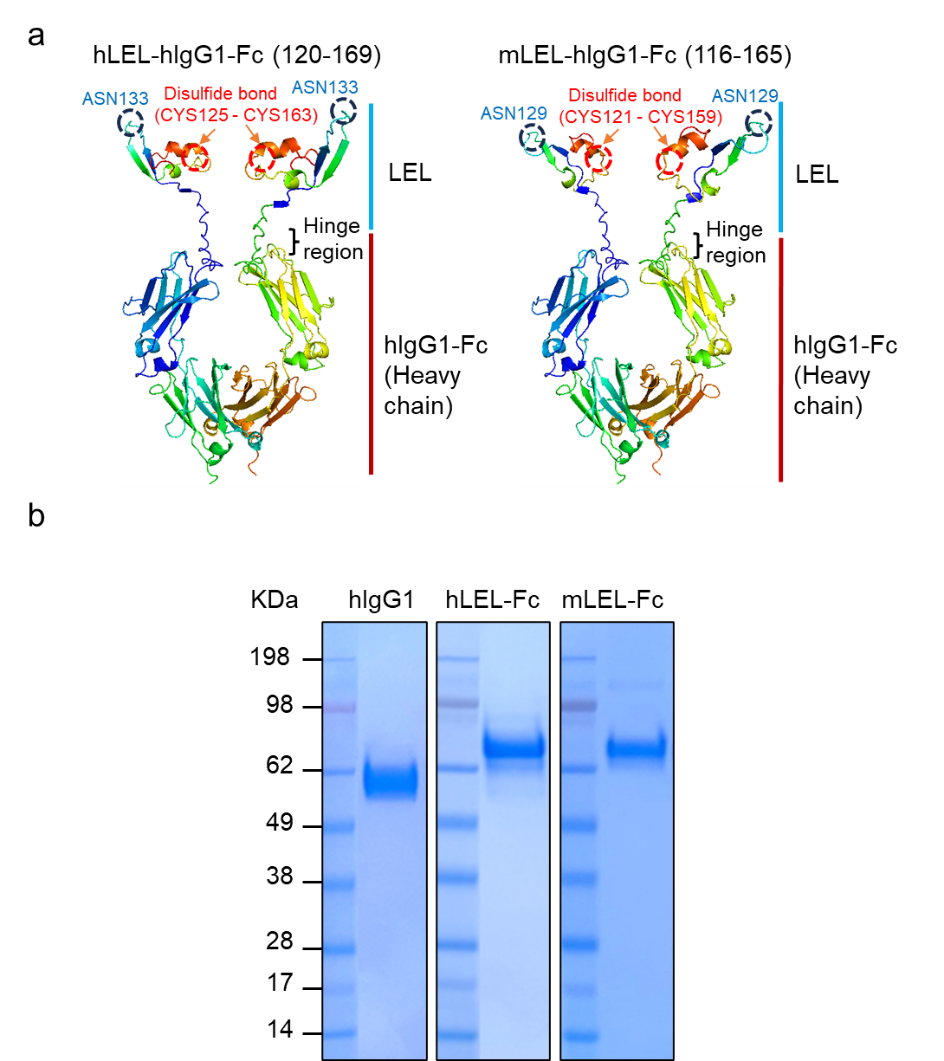
**

**Figure S3. LEL-Fc production.** **a** Structure of human LEL-hIgG1-Fc and mouse LEL-hIgG1-Fc. **b** Representative images of Coomassie blue staining for validation of LEL-Fc fusion protein.

**Figure S4. The incidence of CIA.** The effect of LEL-Fc on the incidence of CIA was analyzed.


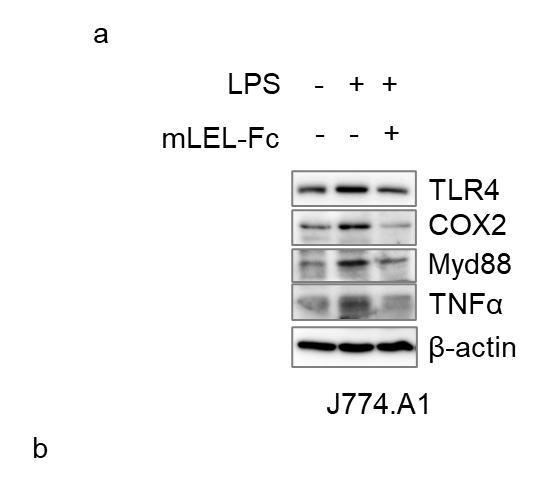


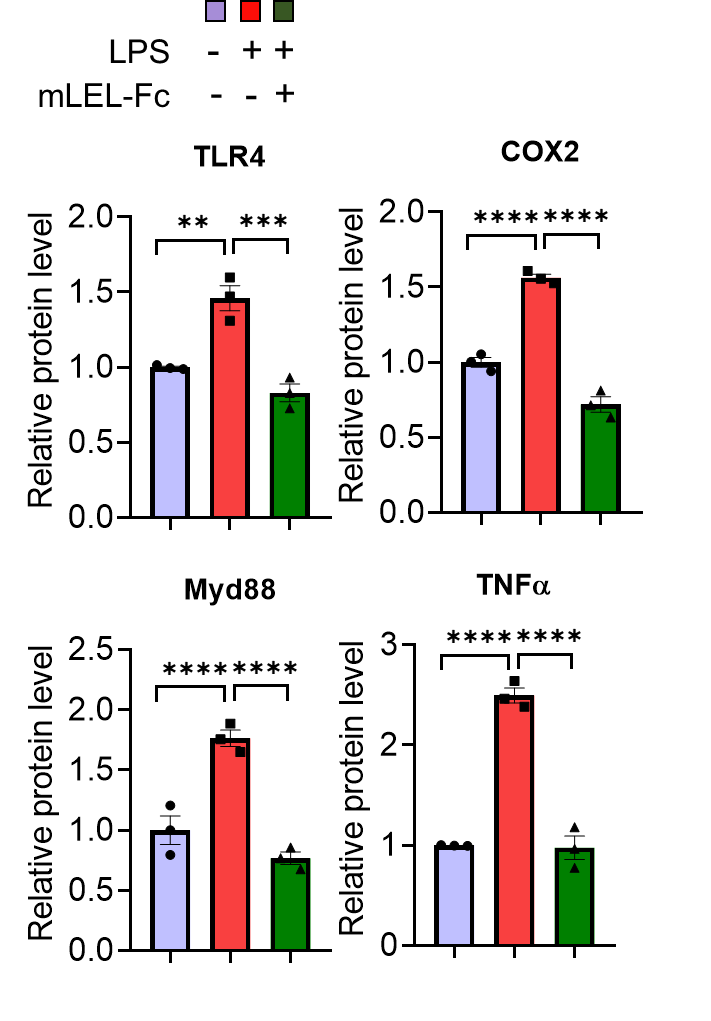


**Figure S5.** **mLEL-Fc suppresses TLR4-mediated inflammatory signaling induced by LPS.** (a) Immunoblot analysis showing the inflammatory TLR4-related downstream signaling pathways in J774 macrophage cells. (b) Quantitative analysis of representative protein expressions related to inflammatory signaling. All the quantitative data were presented as mean ± SD and significance was calculated by one-way ANOVA; **p <0.01, ***p <0.001, ****p <0.0001.


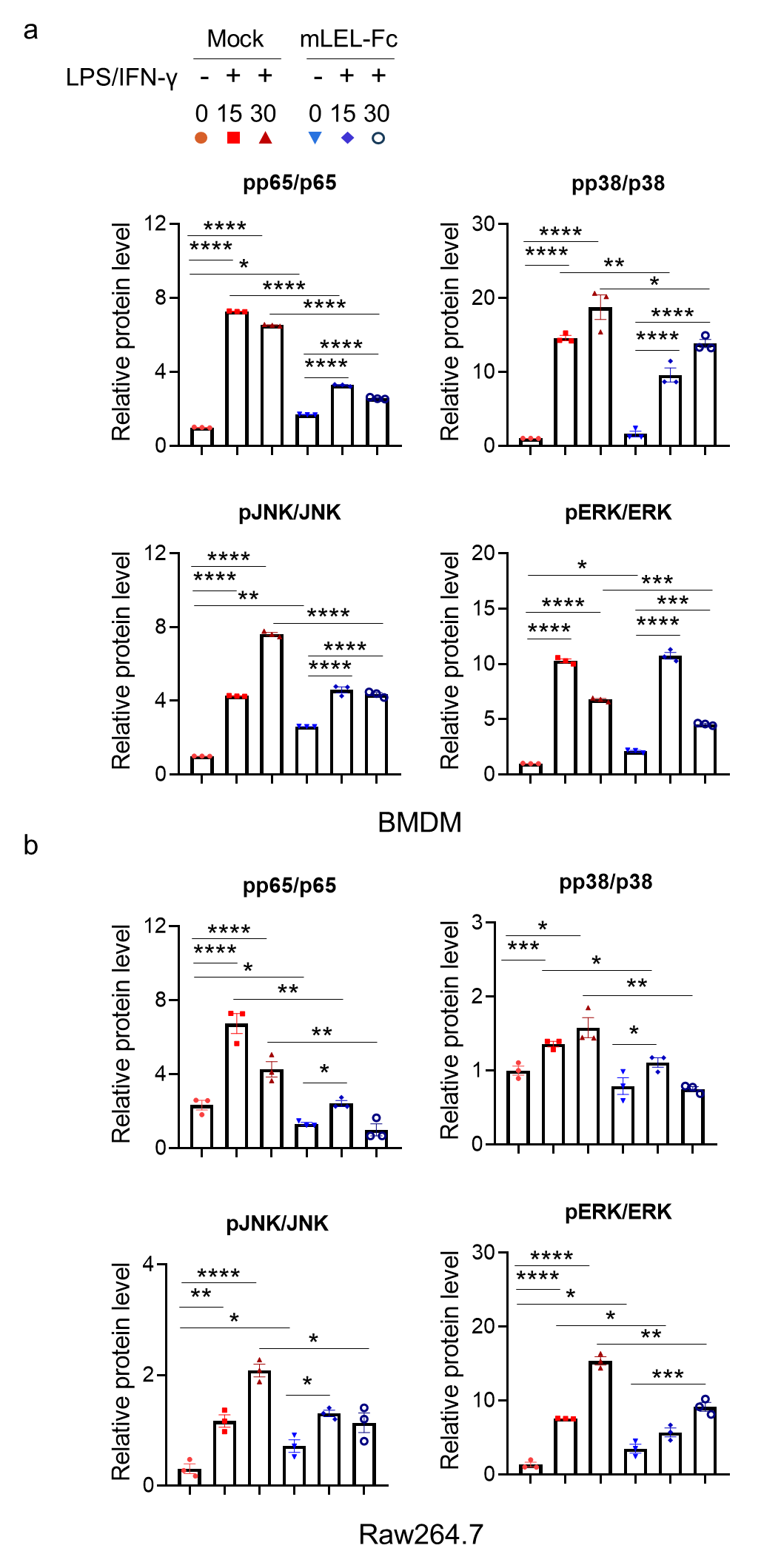


**Figure S6** **Relative protein expression.** Quantification of NF-_K_B and MAPK expression in Figure 4c. All the quantitative data were presented as mean ± SD and significance was calculated by two-way ANOVA; *p <0.05, **p <0.01, ***p <0.001, ****p <0.0001.


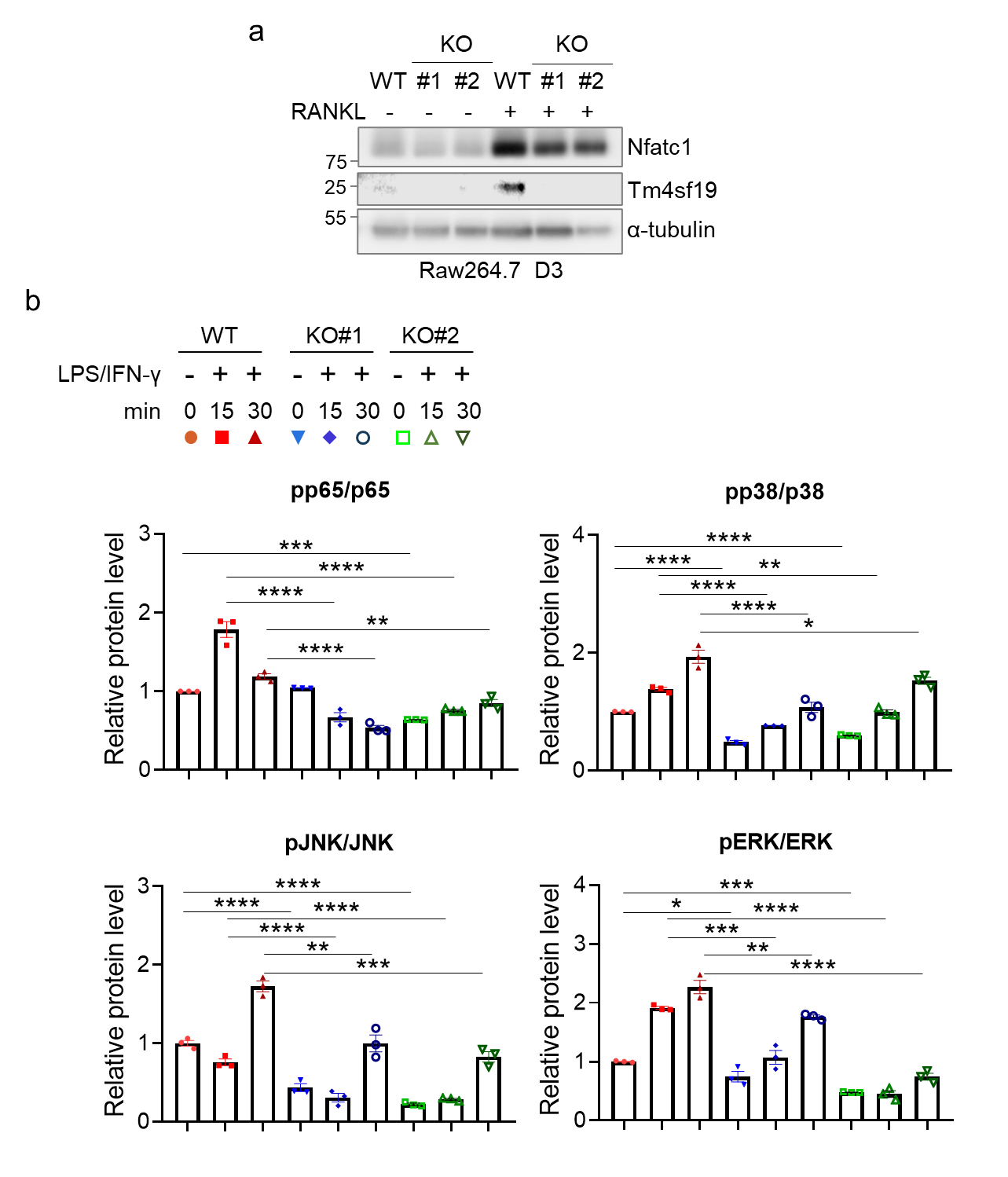


**Figure S7. Relative protein expression induced by LPS/IFN-γ in WT or Tm4sf19 KO Raw264.7 cells.** (a) Protein expression of wild type and Tm4sf19 knock-out clones #1 and #2 was confirmed by western blot after incubating with or without RANKL for 3 days. Nfatc1 was used for osteoclast differentiation markers and α-tubulin was used for a loading control. (b) Quantification of protein expression is analyzed in Figure 4d. All quantitative data are presented as mean ± SD and significance was calculated using two-way ANOVA; *p <0.05, **p <0.01, ***p <0.001, ****p <0.0001.


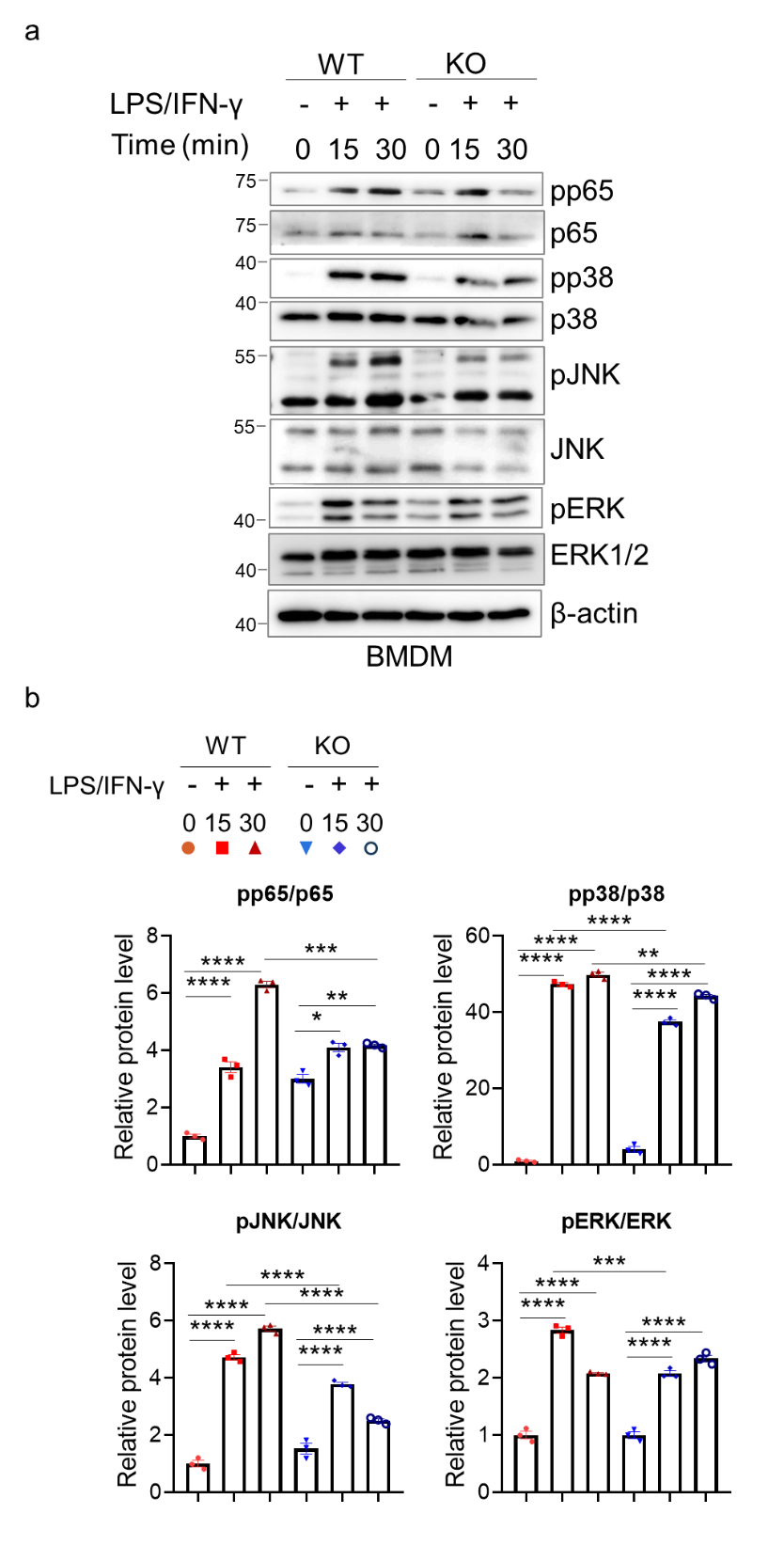


**Figure S8. The effect of Tm4sf19 deficiency on LPS/ IFN-γ-induced inflammatory signaling** **pathways.** (a) Immunoblot results showing that NF-_K_B and MAPK signaling induced by LPS/ IFN-γ treatment at the indicated time points in Tm4sf19 knock out BMDM. (

b) Quantification of protein expression is shown. All quantitative data are presented by mean ± SD, and significance was calculated using two-way ANOVA; *p <0.05, **p <0.01, ***p <0.001, ****p <0.0001.


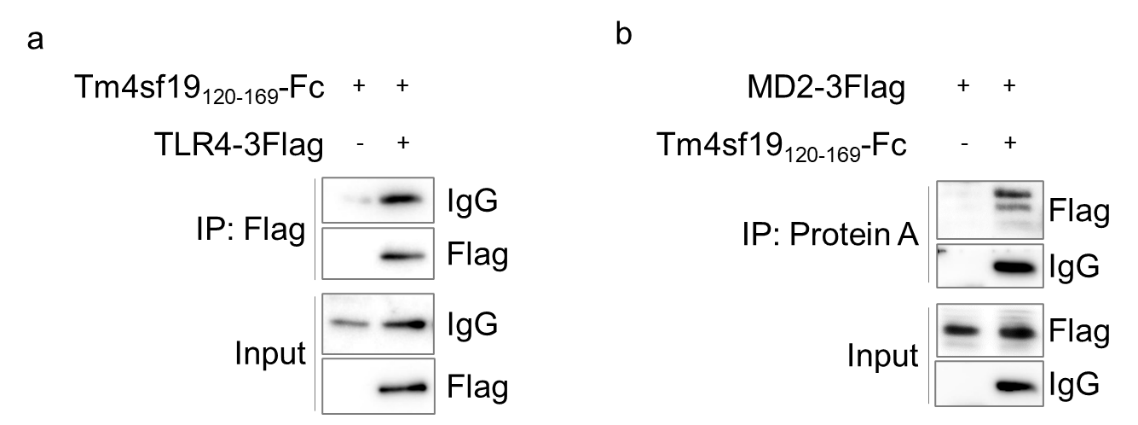


**Figure S9. The interaction of TLR4 or MD2 with Tm4sf19_120-169._** Immunoprecipitation results of the interaction between Tm4sf19_120-169_ and TLR4 (a) or MD2(b)


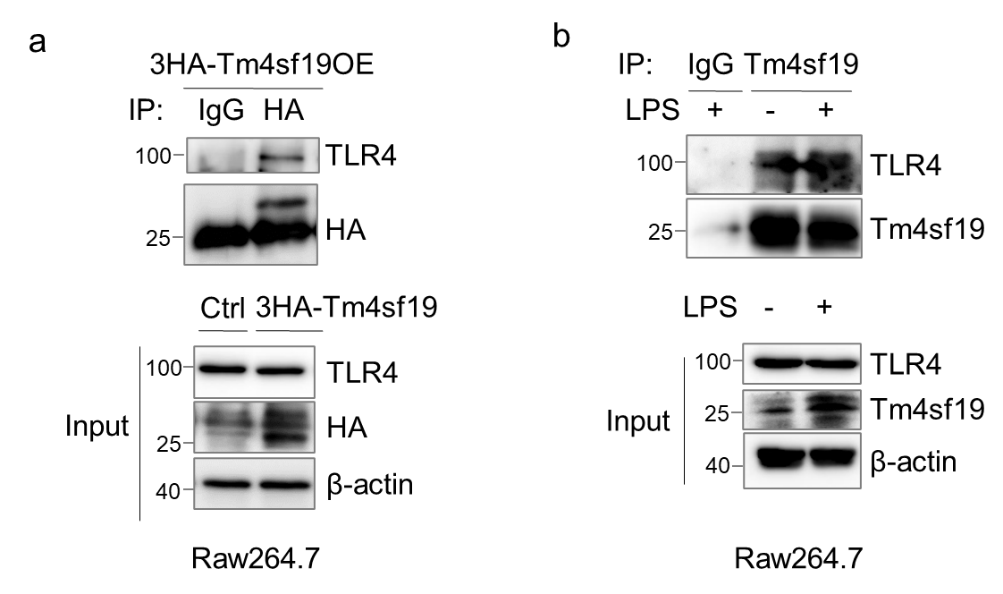


**Figure S10.** **Interaction between Tm4sf19 and TLR4.** Endogenous interaction of Tm4sf19 with TLR4 was studied. **a** Stably Tm4sf19 expressed Raw 264.7 cells were immunoprecipitated with an anti-HA antibody. **b** Immunoprecipitation analysis of Tm4sf19 and TLR4. LPS non-treated or treated Raw264.7 cells were pulled down with a Tm4sf19 antibody.


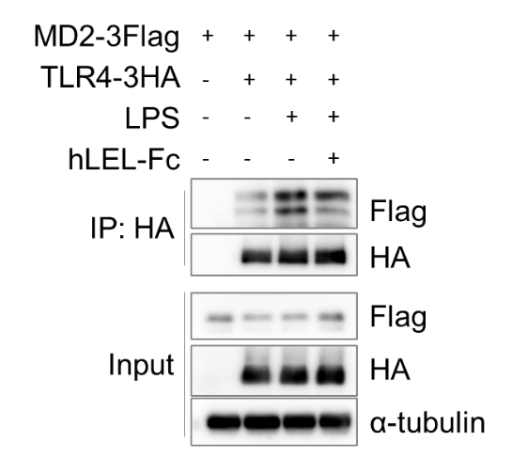


**Figure S11. The effect of hLEL-Fc on TLR4/MD2 complex formation.** Immunoprecipitation results showing the TLR4 and MD2 complex formation with or without LPS.


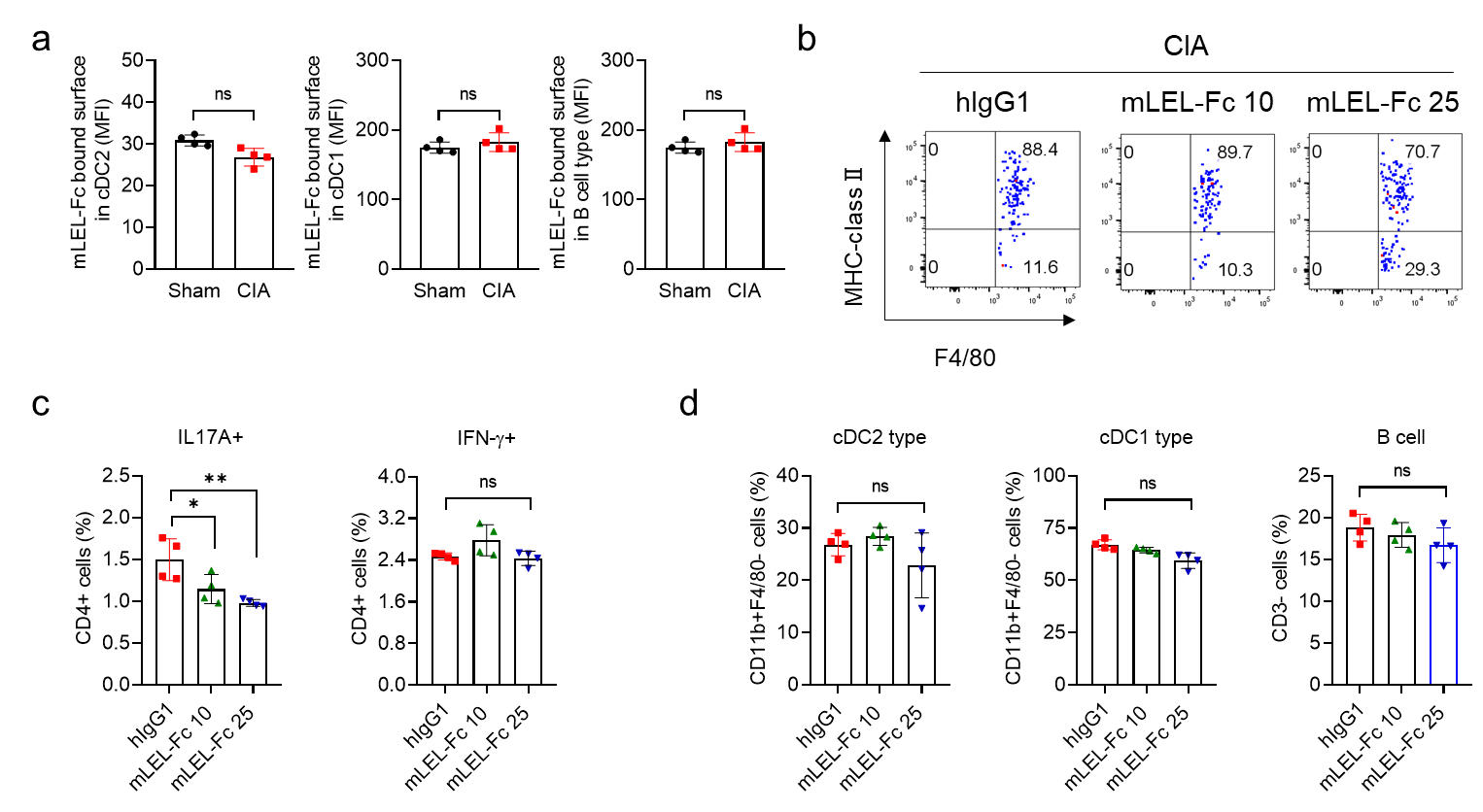


**Figure S12.** **The effect of LEL-Fc on immune phenotyping in spleen of mice with CIA treated with hIgG1 or mLEL-Fc was analyzed.** **a** FACS analysis of the mean fluorescens intensity (MFI) of mLEL-Fc in cDC2, cDC1 and B cells. **b** FACS analysis of M1 macrophage polarization. **c** A representative FACS analysis of IL-17A and IFN-γ after mLEL-Fc treatment. **d** The effect of mLEL-Fc on dendritic cells and B cells.


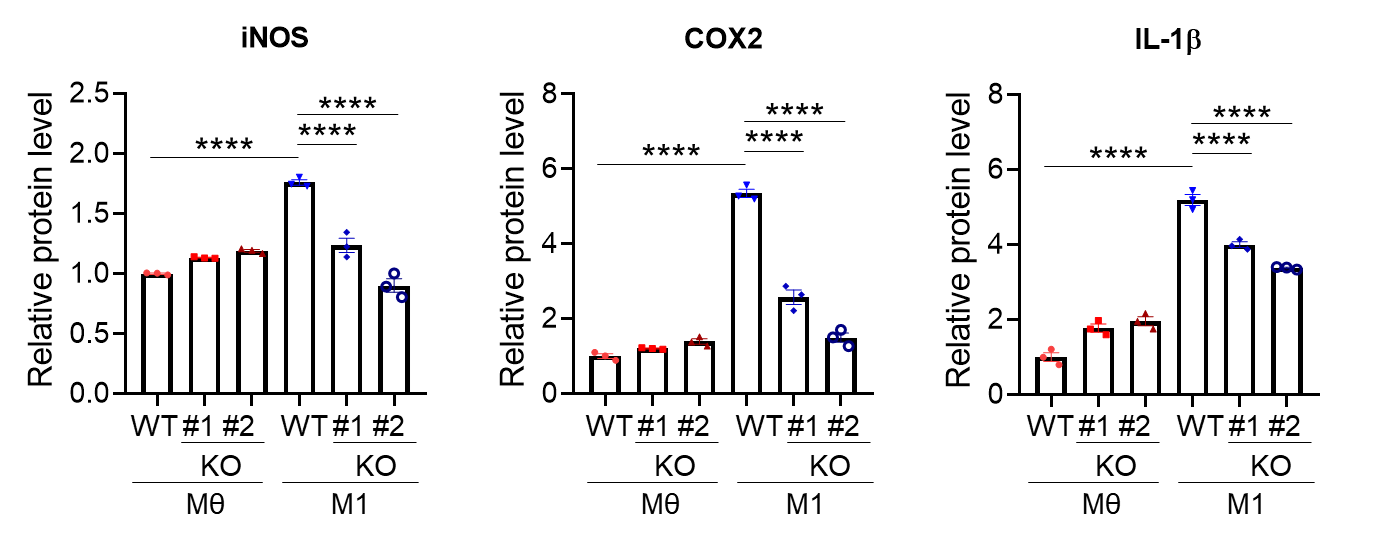


**Figure S13. Relative protein expression.** Quantification of M1 macrophage protein expression in Tm4sf19 knockout clones of Raw264.7 cell lines compared to WT shown in Figure 5g. All quantitative data are presented by meat with ±SD and significance was calculated using two-way ANOVA; ****p <0.0001.


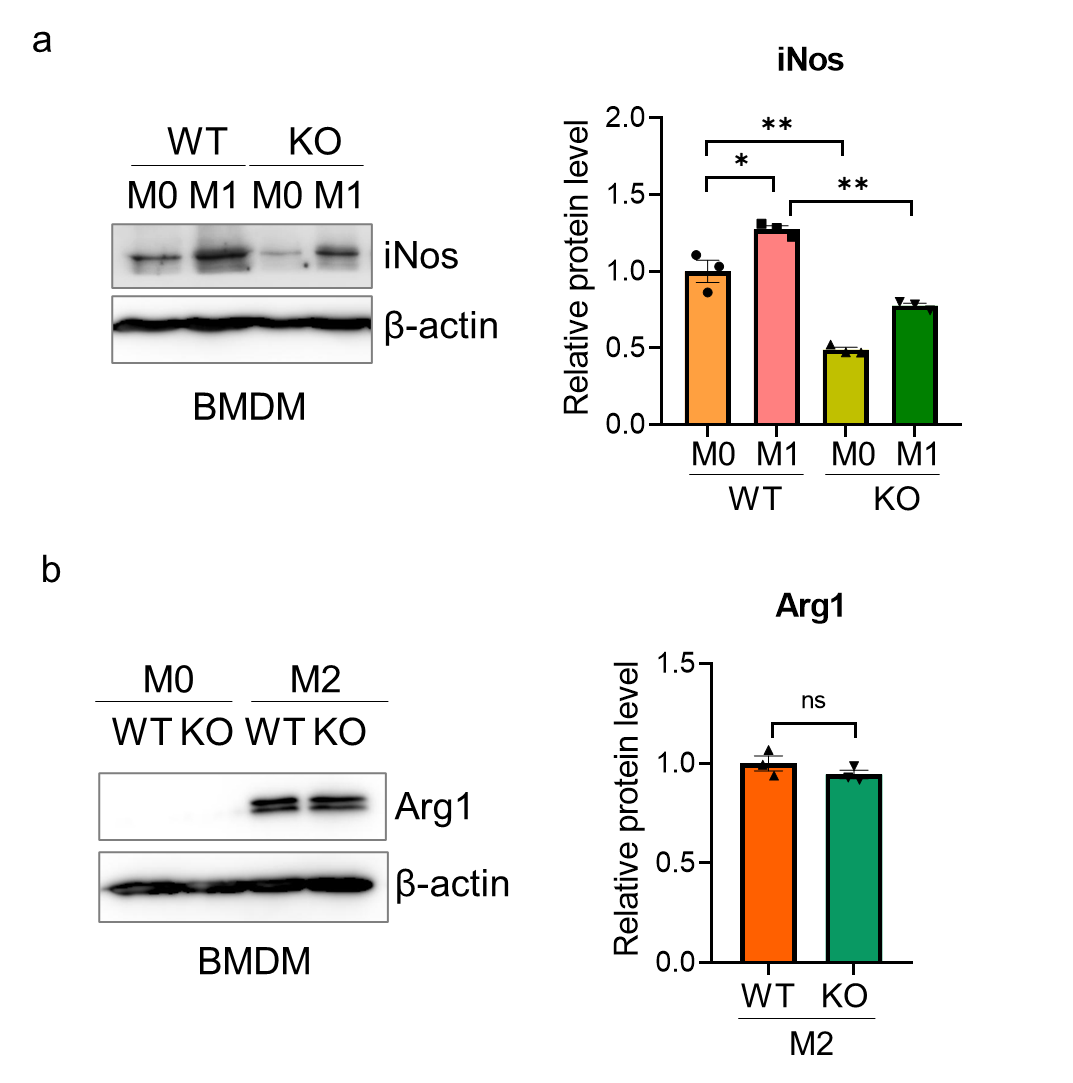


**Figure S14. M1/M2 macrophage differentiation.** Immunoblot analysis was performed with the indicated antibodies after M1 (a) or M2 (b) differentiation of wild-type and Tm4sf19 knockout BMDMs. Quantification of protein expression was performed. All quantitative data are presented by meat with ±SD and significance was calculated using two-way ANOVA(a) or t-test (b); *p <0.05, **p <0.01, ns=no significance.


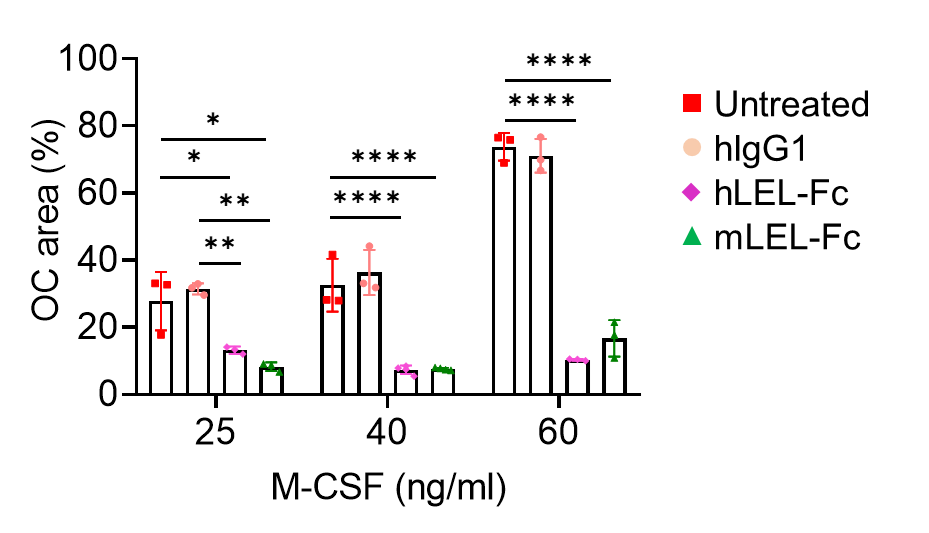


**Figure S15. Percent of osteoclast area per field.** TRAP^+^ osteoclast area (%) was calculated in untreated, hIgG1, hLEL-Fc and mLEL-Fc treated BMDMs during osteoclast differentiation in the Figure 6a. All quantitative data are expressed as mean with ± SD, and significance was calculated using one-way ANOVA; *p <0.05, **p <0.01, ****p <0.0001


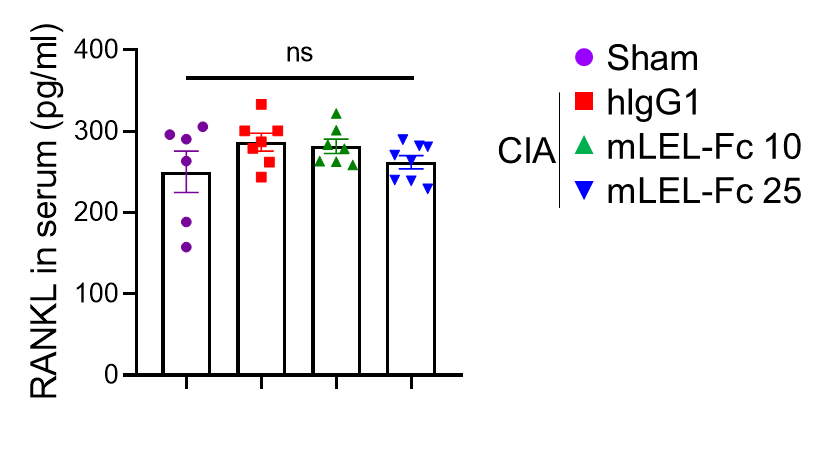


**Figure S16. Serum RANKL production.** RANKL levels in the serum of CIA mice treated with hIgG1, mLEL-Fc or sham mice were measured by ELISA assay. All quantitative data are expressed by mean ± SD, and significance was calculated using one-way ANOVA; ns=no significance.


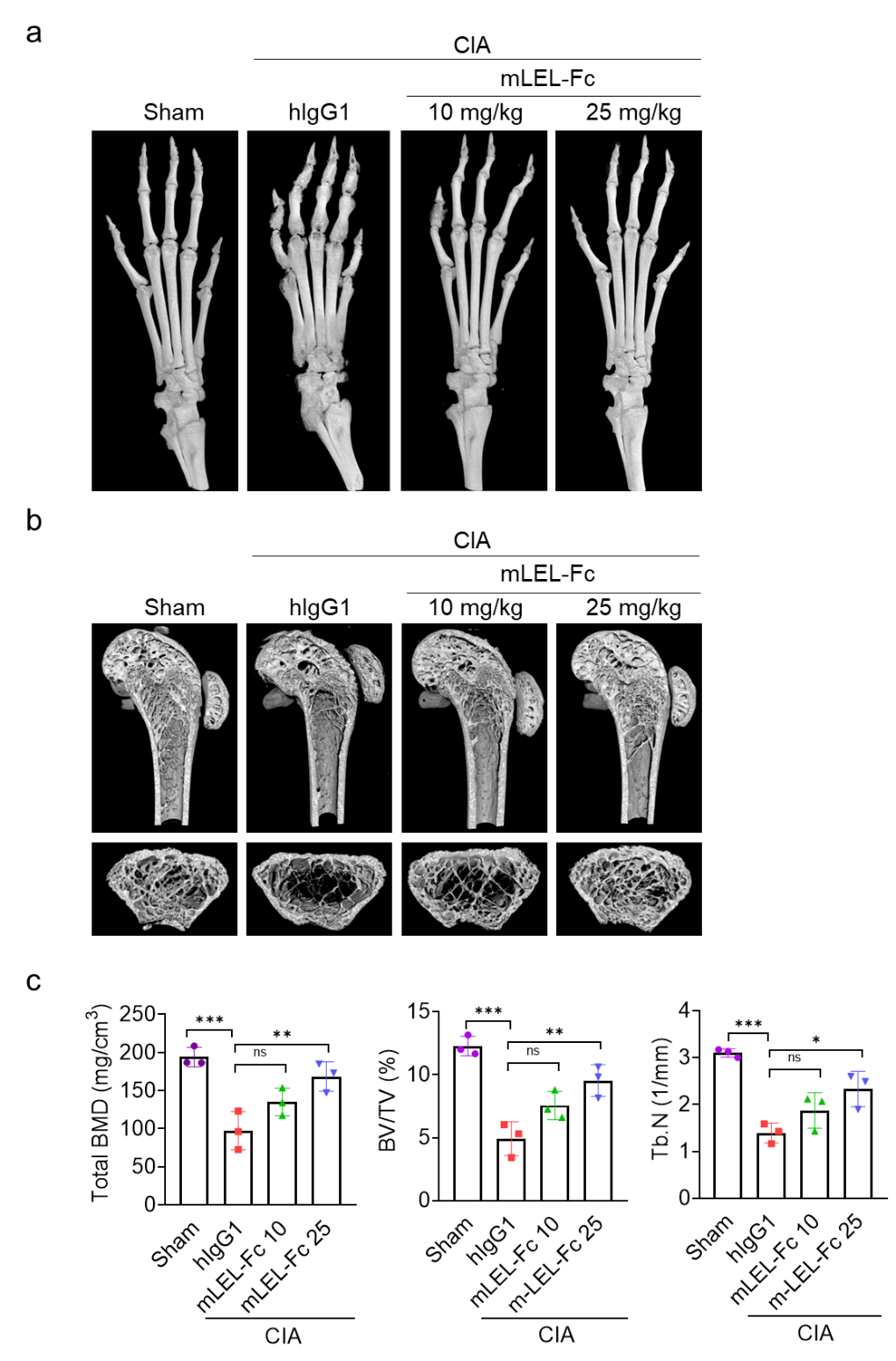


**Figure S17.** **μCT analysis of the hind limb in mice with CIA treated with mLEL-Fc.** **a** Representative μCT images of the rear paw. **b** μCT analysis of LEL-Fc-treated femurs in CIA-induced mice **c**. Total BMD, BV/TV and Tb,N of femurs were analyzed. Statistical analysis was performed with data from three mice. The significance was calculated by one way ANOVA; *p <0.05, **p <0.01, ***p <0.001, ns=no significance.


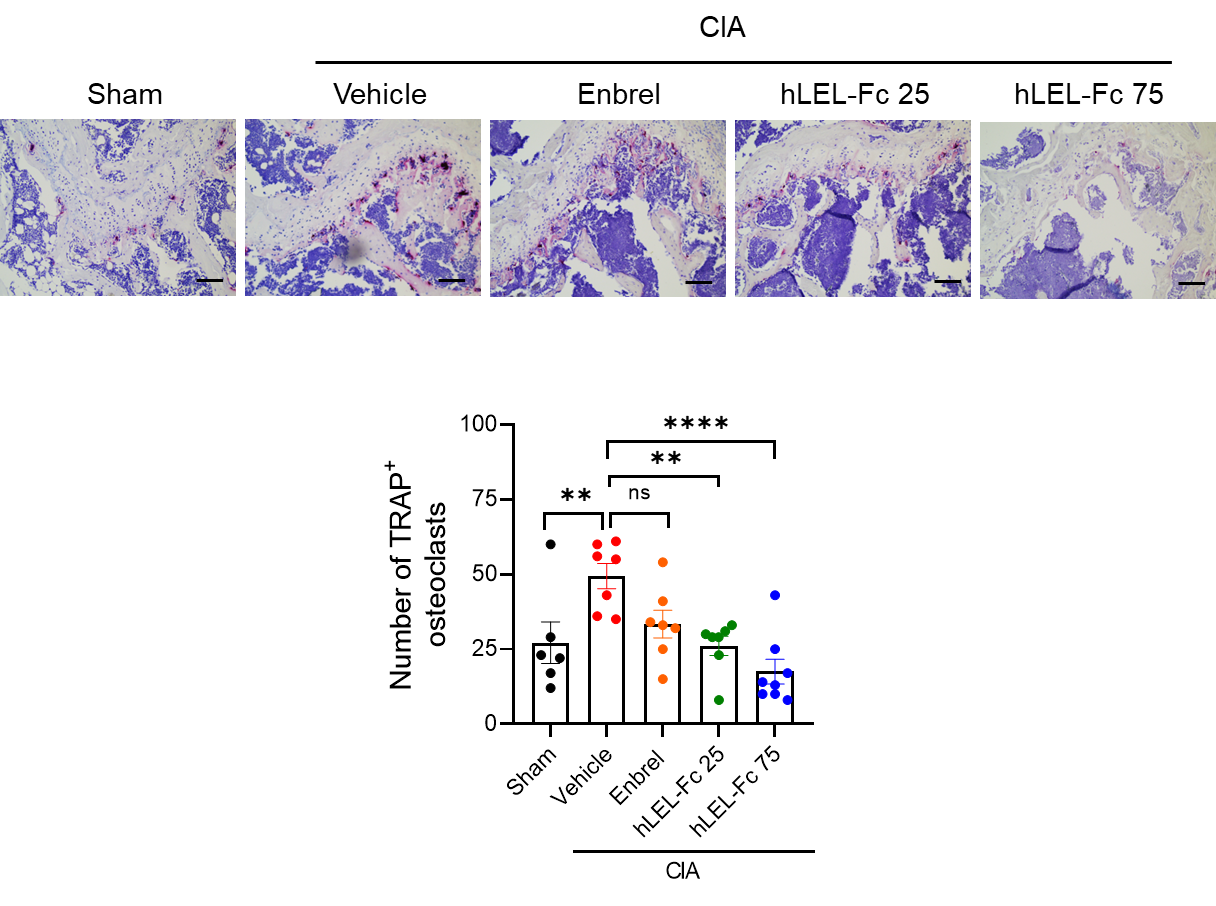


**Figure S18.** **The effect of mLEL-Fc on osteoclast activation in mice with CIA.** Representative photos of femurs stained with TRAP were taken. The number of TRAP^+^ osteoclasts per field was calculated. The significance was calculated by one way ANOVA; **p <0.01, ****p <0.0001, ns=no significance.
