# Supplementary material for: Tm4sf19 inhibition ameliorates inflammation and bone destruction in collagen-induced arthritis by suppressing TLR4-mediated inflammatory signaling and abnormal osteoclast activation
Source: Bone Res. 2025 Mar 24;13:40. doi: 10.1038/s41413-025-00419-y (PMC11933450; doi:10.1038/s41413-025-00419-y)
Supplement: Supplementary file 2 — Supplemental materials and methods [file 41413_2025_419_MOESM2_ESM.docx]

**Tm4sf19 inhibition ameliorates inflammation and bone destruction in collagen-induced arthritis by suppressing TLR4-mediated inflammatory signaling and abnormal osteoclast activation**

Sujin Park^1^, Kwiyeom Yoon^2^, Eunji Hong^1^, Min Woo Kim^2^, Min Gi Kang^1^, Seiya Mizuno^3^, Hye Jin Kim^2^, Min-Jung Lee^2^, Hee Jae Choi^2^, Jin Sun Heo^1^, Jin Beom Bae^2^, Haein An^1^, Naim Park^2^, Hyeyeon Park^1,4^, Pyunggang Kim^1^, Minjung Son^1,4^, Kyoungwha Pang^1^, Je Yeun Park^1^, Satoru Takahashi^5^, Yong Jung Kwon^1^, Dong-Woo Kang^2^, and Seong-Jin Kim^1,2*^

^1^GILO Institute, GILO Foundation, Seoul, Republic of Korea, ^2^Medpacto Inc., Seoul, Republic of Korea, ^3^Laboratory Animal Resource Center in Transborder Medical Research Center, Institute of Medicine, University of Tsukuba, Tsukuba, Japan, ^4^Department of Biological Sciences, Sungkyunkwan University, Suwon, Republic of Korea, and ^5^Department of Anatomy and Embryology, Faculty of Medicine, University of Tsukuba, Tsukuba, Japan.

**Antibodies**

Following antibodies were used. Anti-IL-17A (79056, abcam, 1:1000), anti-IL-6 (290735, abcam, EPR23819-103, 1:1000), anti-IL-1β (9722, abcam, 1:1000), anti-TNF-α (6671, abcam, 1:1000), anti-TLR4 (293072, Santa Cruz Biotechnology, 25, 1:1000), anti-Myd88(74532, Santa Cruz Biotechnology, E-11, 1:1000), anti-iNOS (178945, abcam, EPR16635, 1:1000), anti-Arg1 (124917, abcam, EPR6671(B), 1:1000), anti-COX2 (35-8200, Invitrogen, COX299, 1:1000), anti-F4/80 (377009, Santa Cruz Biotechnology, C-7, 1:1000), anti-β-actin (A5441, Sigma Aldrich, AC-15, 1:5000), anti-α-tubulin (T5168, Sigma Aldrich, B-5-1-2, 1:5000), anti-Tm4sf19 (customized), anti-HA (7392, Santa Cruz Biotechnology, F-7, 1:1000), anti-Flag (F-3165, Sigma Aldrich, M2, 1:1000), anti- human IgG1 Fc Secondary antibody, HRP (A-10648, Thermo Fisher, 1:1000), anti-GST(138, Santa Cruz Biotechnology, B-14, 1:1000), anti-phospho-pERK1/2 (9101, Cell Signaling Technology, Thr202/Tyr204, 1:1000), anti-ERK (9102, Cell Signaling Technology, 1:1000), anti-phospho-p65 (3033, Cell Signaling Technology, 93H1, 1:1000), anti-p65 (8242, Cell Signaling Technology, D14E12, 1:1000), anti-phospho-p38, 9211, Cell Signaling Technology, 1:1000), anti-p38 (9212, Cell Signaling Technology, 1:1000), anti-phospho-pJNK (4668, Cell Signaling Technology, 81E11, 1:1000), anti-pJNK (9252, Cell Signaling Technology, 1:1000), anti-Nfatc1 (7294, Santa Cruz Biotechnology, 7A6, 1:1000).

**Transient or stable cell line generation.**

Full length or deletion mutants of Tm4sf19 constructs from a previous report were used ^1^. Full length of TLR4 was inserted into pCS4-3HA vector or pCS4-3Flag and full length of MD2 was inserted pCS4-3Flag. For transient expression, DNA constructs were co-transfected using PEI reagent in 293T or HEK293 cells. For Tm4sf19 expressing stable cell line, Lenti viral system was used as described in a previous report.^1^

**Fluorescence-Activated Cell Sorting (FACS) analysis**

Spleens were harvested and pressed through a 100-μm nylon strainer with the plunger end of syringe for single-cell suspension. Red blood cells were lysed with ACK lysis buffer (1.55 mM NH_4_Cl / 12 mM NaHCO_3/_ 1 mM EDTA). After filtered through a 40 μm nylon strainer, resuspended in a buffer supplemented with FACS buffer (2% FBS/ 1mM EDTA/ 1X PBS). Cells were blocked with anti-CD16/32 (Biolegend, San Diego, CA) and stained for 30 min with conjugated antibodies obtained from Biolegend. The following mAbs were used for staining: BV421–conjugated mAbs to MHC class II (M5/114.15.2); BV510- conjugated mAbs to CD45 (30-F11); PE–conjugated mAb to CD11b (M1/70) and PerCP–conjugated mAbs to F4/80 (BM8). Viability Dye 780 (BioGems™) was used to exclude dead cells. Mouse LEL-Fc and Isotype-matched hIgG1 control were labelled by Alexa Fluor™ 647 Antibody Labeling Kit (Thermo Fisher Scientific, Waktham, MA). Labeled cells were analyzed by BD FACS Canto II (BD Biosciences, Franklin Lakes, NJ), and data were analyzed using FlowJo (BD Bioscience, ver. 10.8) software.

**LEL-Fc fusion protein preparation**

Mouse LEL-Fc, human LEL-Fc or hIgG1-Fc fusion protein was obtained as described in previous report.^1^ Briefly, constructs were transfected with ExpiFectamine™ CHO transfection kit (Gibco™, A29129). Cells were harvested and centrifuged to collect supernatant. Fc-fusion protein was purified using the protein A column (Cytiva, XK26 column) and analyzed by SDS-PAGE gel in non-reducing condition.

1 Park, S. *et al.* Tm4sf19 deficiency inhibits osteoclast multinucleation and prevents bone loss. *Metabolism* **151**, 155746, doi:10.1016/j.metabol.2023.155746 (2024).
